# Genetic and Pharmacological Inhibition of Astrocytic Mysm1 Alleviates Depressive‐Like Disorders by Promoting ATP Production

**DOI:** 10.1002/advs.202204463

**Published:** 2022-11-22

**Authors:** Heyang Zhang, Shuirong Liu, Qiaozhen Qin, Zhenhua Xu, Yannv Qu, Yadi Wang, Jianing Wang, Zhangzhen Du, Shanshan Yuan, Shunming Hong, Zhilin Chang, Wenyan He, Xinlong Yan, Yiran Lang, Rongyu Tang, Yan Wang, Lingling Zhu, Xiaoxia Jiang

**Affiliations:** ^1^ Beijing Institute of Basic Medical Sciences 27 Taiping Road, Haidian District Beijing 100850 China; ^2^ Faculty of Environmental and Life Sciences Beijing University of Technology Beijing 100124 China; ^3^ Department of Geriatrics Peking University Shenzhen Hospital Shenzhen Guangzhou 518036 China; ^4^ China National Clinical Research Center for Neurological Diseases Jing‐Jin Center for Neuroinflammation Beijing Tiantan Hospital Capital Medical University Beijing 100050 China; ^5^ Beijing Innovation Center for Intelligent Robots and Systems Beijing Institute of Technology Beijing 100081 China; ^6^ Anhui Medical University Hefei Anhui 230032 China; ^7^ Co‐innovation Center of Neuroregeneration Nantong University Nantong 226019 China

**Keywords:** antidepressant effect, aspirin, astrocyte, ATP, Mysm1

## Abstract

Major depressive disorder (MDD) is a leading cause of disability worldwide. A comprehensive understanding of the molecular mechanisms of this disorder is critical for the therapy of MDD. In this study, it is observed that deubiquitinase Mysm1 is induced in the brain tissues from patients with major depression and from mice with depressive behaviors. The genetic silencing of astrocytic Mysm1 induced an antidepressant‐like effect and alleviated the osteoporosis of depressive mice. Furthermore, it is found that Mysm1 knockdown led to increased ATP production and the activation of p53 and AMP‐activated protein kinase (AMPK). Pifithrin α (PFT α) and Compound C, antagonists of p53 and AMPK, respectively, repressed ATP production and reversed the antidepressant effect of Mysm1 knockdown. Moreover, the pharmacological inhibition of astrocytic Mysm1 by aspirin relieved depressive‐like behaviors in mice. The study reveals, for the first time, the important function of Mysm1 in the brain, highlighting astrocytic Mysm1 as a potential risk factor for depression and as a valuable target for drug discovery to treat depression.

## Graphical abstract

Deubiquitinase Mysm1 is induced in the brain tissues from patients with major depression and from mice with depressive behaviors. Genetic and pharmacological inhibition of Mysm1 lead to increased ATP production and activation of p53 and AMP‐activated protein kinase (AMPK) and then relieved depressive‐like behaviors in mice. This study reveals the important function of Mysm1 in the brain.

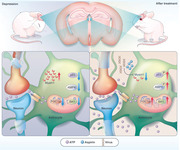

## 1 Introduction

Depression is a common mental disorder and a leading cause of disability worldwide. The associated direct and indirect costs of major depression are set to pass US $6 trillion by 2030.^[^ ^1^ ^]^ The core symptoms of major depression include depressive mood, decreased drive, loss of interest and pleasure.^[^ ^2^, ^3^ ^]^ In addition, there is a higher liability for osteoporosis in patients with major depression.^[^ ^4^, ^5^ ^]^ Conventional antidepressants are associated with substantial variations in clinical treatment efficacy, and newly developed drugs show a high failure rate in clinical trials.^[^ ^6^ ^]^ The limited success of drug discovery in the context of depression is ultimately linked to an inadequate understanding of the underlying neurobiological mechanisms of this disorder and the inadequacies of the diagnosis. Therefore, detailed mechanistic investigations are urgently needed.

Numerous studies have revealed that the abnormal neuronal function, overactivated microglia, and excessive neuroinflammatory reactions are involved in the onset of depression.^[^ ^7^, ^8^ ^]^ Emerging evidences have now implicated astrocyte dysfunction in the pathogenesis of MDD.^[^ ^9^ ^]^ Activated astrocytes produce pro‐inflammatory cytokines such as interleukin IL1*β*, TNF*α*, which are key to the induction of depressive symptoms. As the most abundant glial cell type in the mammalian brain, astrocytes are reported to secret ATP for astrocyte‐neuron communications. Astrocytes are reported to secret ATP through several pathways and the decreased ATP concentration has been confirmed to be involved in depression‐like disorders.^[^ ^10^ ^]^ However, in spite of all the investigations, the mechanism of astrocytic ATP release is still far from clear.

Mysm1 is a deubiquitinase comprising the SANT, SWIRM, and MPN domains that cleaves monoubiquitin at K119 of histone H2A and then epigenetically regulates the expression of transcription factors that are essential for cell development. In addition, Mysm1 can remove M1, K6, K27, and K63‐polyubiquitin chains and then modulate downstream events.^[^ ^11^, ^12^, ^13^ ^]^ The characterization of Mysm1‐deficient mice and patients with Mysm1 mutations highlights the important function of Mysm1 in hematopoiesis, immunity, and other aspects of mammalian physiology. Mysm1‐deficient mice exhibited growth retardation, skeletal deficits, and complex hematopoietic and immune dysfunctions.^[^ ^14^, ^15^, ^16^ ^]^ Patients with Mysm1 mutations demonstrated anemia and leukopenia and, in some cases, mutations were associated with growth retardation.^[^ ^17^ ^]^ Bahrami et al. reported that two patients with Mysm1 mutations showed complex developmental aberrations, including mild skeletal anomalies, neurocognitive developmental delay, and cataracts.^[^ ^15^ ^]^ However, the precise role of Mysm1 in the central nervous system has not yet been investigated.

In this study, we revealed that Mysm1 was significantly upregulated in the brain tissues from patients with major depression and mice with depressive‐like behaviors. The knockdown of astrocytic Mysm1 relieved murine depressive‐like behaviors and alleviated the osteoporosis of depressive mice. Increased activation of the p53/AMPK pathway and ATP production was found in astrocytes with Mysm1 knockdown. Aspirin was found to inhibit Mysm1 expression, and the injection of aspirin showed antidepressant effects. Together, these results provide evidence that there is a distinct role for Mysm1 in regulating astrocytic function in depressive disorders. The role of both genetic and pharmacological agents in the inhibition of astrocytic Mysm1 to alleviate depressive‐like disorders suggests that astrocytic Mysm1 may be a valuable target for drug discovery in depression.

## 2 Results

### 2.1 Mysm1 was Increased in the Brain Tissues from Mice with Depressive‐Like Behaviors and from Patients with Major Depression

**Figure 1:**
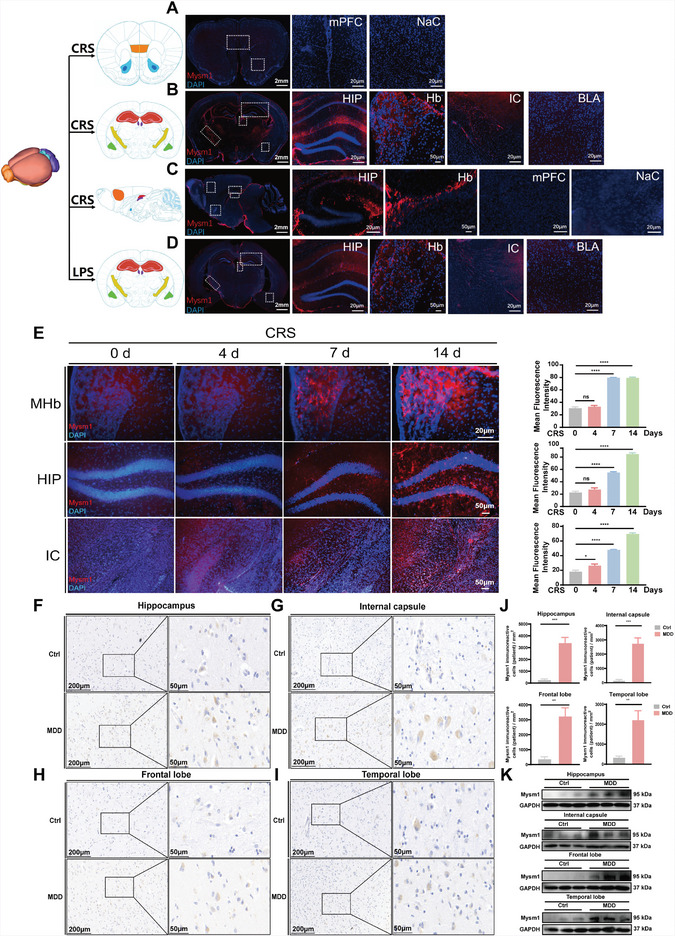
Mysm1 was increased in the brain tissues from mice with depressive‐like behaviors and from patients with major depression. A,B) Immunofluorescent staining of coronal brain sections in the mPFC, NaC, HIP, Hb, IC and BLA of CRS mice. Scale bars, 2 mm, 100 µm, 50 µm, 20 µm. C) Immunofluorescent staining of sagittal brain sections in the HIP, Hb, mPFC, and NaC of CRS mice. Scale bars, 2 mm, 100 µm. D) Immunofluorescent staining of coronal brain sections in the HIP, Hb, IC and BLA of LPS mice. Scale bars, 2 mm, 100 µm, 50 µm, 20 µm. E) Immunofluorescent staining and quantitative analysis in the MHb, HIP and IC of CRS mice at 0, 4, 7, and 14 days. Scale bars, 50 µm, 20 µm. F–I) Mysm1 expression levels in the hippocampus, internal capsule, frontal lobe, temporal lobe from elderly human patients with MDD (*n* = 4) and control patients (*n* = 4) examined by immunohistochemical analysis. Scale bars, 200 µm, 50 µm. J) Quantitative analysis of Mysm1 positive cells in immunohistochemical staining in the hippocampus, internal capsule, frontal lobe, temporal lobe. K) Mysm1 expression levels in the hippocampus, internal capsule, frontal lobe, temporal lobe from elderly human patients with MDD (*n* = 4) and control patients (*n* = 4) examined by western blot analysis. Data represent the mean ± SEM; ns, not significant; *p < 0.05, **p < 0.01, ***p < 0.001 and ****p < 0.0001.

To examine whether Mysm1 may play a role in the pathophysiology of depression, we exposed male C57BL/6N mice to 2 weeks of chronic restraint stress (CRS) to mimic depression.^[^ ^18^ ^]^ Depressive‐like phenotypes in CRS mice were validated by the longer immobility time in the tail suspension test (TST) and forced swimming test (FST) and a lower score in the coat score (CS) assay (Figure S1, Supporting Information). The murine brain was sectioned into coronal or sagittal planes, and slices were collected for staining. Immunostaining data showed that the Mysm1 protein was highly expressed in the medial habenula (MHb), hippocampus (HIP), and internal capsule (IC) of CRS mice (**Figure** **1**A–C), whereas there was no evident expression of Mysm1 in their control counterparts (Figure S2A, Supporting Information). The lateral habenula (LHb) is an important brain region in depression control. In contrast to the MHb region, there was almost no detectable expression in the LHb from both CRS mice and the control mice. Interestingly, there was no evident expression in classical depression nuclei, such as the medial prefrontal cortex (mPFC), nucleus accumbens (Nac), and basolateral amygdaloid nucleus (BLA) (Figure 1A–C). To test whether Mysm1 upregulation is universal in depression, we examined mice in which depression was induced by treatment with lipopolysaccharide (LPS). Ten days of intraperitoneal LPS treatment (0.5 mg kg^−1^ per day, i.p.) caused a strong depressive‐like phenotype in mice, as confirmed by the TST, FST, and CS assays (Figure S1, Supporting Information). As expected, the mice with LPS‐induced depression also showed significant Mysm1 upregulation in the MHb, HIP, and IC compared with their saline group (Figure 1D; Figure S2B, Supporting Information). Notably, the increase in Mysm1 protein was not evident in the mice subjected to four days of chronic restraint stress. With the extension of the chronic restraint time, the expression of Mysm1 gradually increased in the MHb, HIP, and IC (Figure 1E).

To further explore the possible role of Mysm1 in human brain function, postmortem brain tissues from elderly patients with or without major depression were collected for immunohistochemical and western blot analysis. Immunohistochemical staining and the corresponding quantitative analysis data showed that, compared with those from patients without depression (Ctrl), there were more Mysm1 positive cells in the hippocampus, internal capsule, frontal lobe, and temporal lobe brain sections from patients with severe depression (MDD) (Figure 1F–J and **Table** **1**). Western blot data further confirmed the high expression of Mysm1 in the brain tissues from patients with MDD (Figure 1K).

**Table 1:** Patient demography showing gender, age, PMD, cause of death and Mysm1 immunoreactivity

						Mysm1 immunoreactivity [Percentage of Mysm1+ cells]			
Group		Gender	Age	PMD[h]	Cause of death	Hippocampus	Internal capsule	Frontal lobe	Temporal lobe
MDD	1	Female	86	23	Renal failure	47.6	58.5	55.7	52.7
	2	Male	72	39	Myocardial infarction	40.6	39	46.6	43.7
	3	Female	85	6	Heart failure	49.2	27.5	43.2	53.8
	4	Female	89	4.5	Cardiogenic shock	44.3	52.1	32.7	51.3
CON	1	Male	89	4.5	Sudden death	26.1	9.1	35.1	35.9
	2	Male	79	4	Pancreatic cancer	32.8	26.4	11.2	32.3
	3	Female	87	5	Pulmonary fibrosis	16.1	32.5	29.5	14.2
	4	Female	85	3.3	Lung cancer	34.5	22.7	22.9	41.6

Table 1 lists the patient demography in the experiment.

Abbreviation: Mysm1, Myb like SWIRM and MPN domains 1. MDD, Major depressive disorder. CON, Elderly people without major depressive disorder.

### 2.2 Mysm1 Knockdown Relieved Depressive‐Like Behaviors and Alleviated the Osteoporosis of Depressive Mice

**Figure 2:**
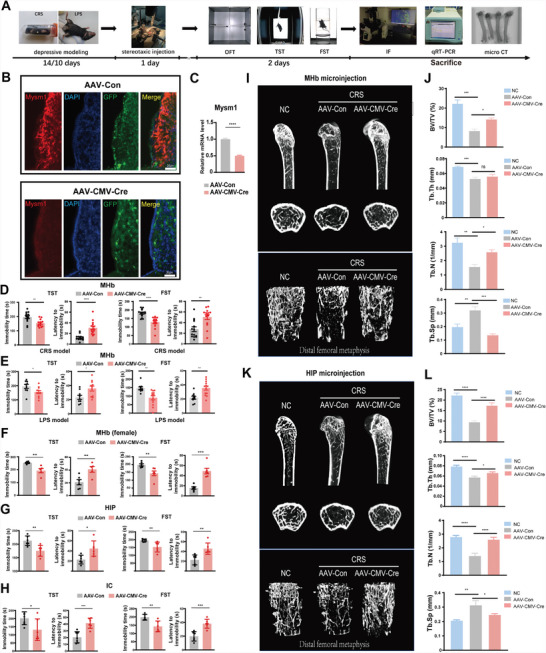
Mysm1 knockdown alleviates depression‐like behaviors and murine osteoporosis. A) The experimental paradigm of virus infection for the treatment of depressive mice after CRS and LPS exposure. B) Immunofluorescence staining and C) qRT‐PCR results of Mysm1 in the MHb of AAV‐CMV‐Cre knockdown depressive mice. Scale bars, 20 µm, Behavioral effects of Mysm1 knockdown in the MHb of D) the CRS mouse model and E) the LPS mouse model in the TST and FST. F) Behavioral effects of Mysm1 knockdown in the MHb of the female CRS mouse model in the TST and FST. Behavioral effects of Mysm1 knockdown in the G) HIP and H) IC of the CRS mouse model in the TST and FST. I) 3D reconstruction results of µCT analysis of femoral trabecular bone in depressive mice (*n* = 4) after down‐regulation of Mysm1 in MHB. J) Analysis of µCT data showing BV/TV, Tb.N, Tb.Th and Tb.Sp in depressive mice after down‐regulation of Mysm1 in MHb. K) 3D reconstruction results of µCT analysis of femoral trabecular bone in depressive mice (*n* = 4) after down‐regulation of Mysm1 in HIP. L) Analysis of µCT data showing BV/TV, Tb.N, Tb.Th and Tb.Sp in depressive mice after down‐regulation of Mysm1 in HIP. Data represent the mean ± SEM; ns, not significant; *p < 0.05, **p < 0.01, ***p < 0.001 and ****p < 0.0001.

To determine whether the knockdown of Mysm1 could alleviate depressive phenotypes, several strategies were devised. Male Mysm1 floxed homozygous mice (Mysm1^fl/fl^) were exposed to two weeks of CRS, and then adeno‐associated viruses (AAV) expressing Cre recombinase and GFP (AAV‐CMV‐Cre) or expressing GFP only (AAV‐Con) under the cytomegalovirus (CMV) promoter were microinjected into the MHb region. Details of virus vectors and injection site are shown in Figure S3 (Supporting Information). Three weeks later, immunostaining, qRT–PCR analysis, and murine behavioral tests were performed (**Figure** **2**A). GFP expression was obvious in the MHb, indicating successful injection (Figure S3B, Supporting Information). Immunofluorescence data demonstrated that AAV‐Con injection did not affect the high expression of Mysm1 in the MHb (Figure 2B). As expected, a marked decrease in Mysm1 expression was observed in the MHb after AAV‐CMV‐Cre injection (Figure 2B). qRT‐PCR data confirmed the reduced Mysm1 level in the MHb (Figure 2C). Accordingly, compared to the controls, Mysm1^fl/fl^ mice with Mysm1 knockdown in MHb showed a significant decrease in immobility duration during TST and FST analysis (Figure 2D). As expected, the mice exposed to LPS treatment also exhibited alleviated depressive‐like behaviors with Mysm1 knockdown in the MHb (Figure 2E). In addition to the male mice, as shown in Figure 2F, the knockdown of Mysm1 alleviated depressive‐like behaviors in female Mysm1^fl/fl^ mice exposed to CRS. To explore whether the knockout of Mysm1 expression in the HIP or IC could induce similar antidepressant effects, AAV‐CMV‐Cre was injected into the CA1 region of the HIP or IC. qRT–PCR data confirmed the reduced Mysm1 level in the HIP or IC injected with AAV‐CMV‐Cre (Figure S4A,B, Supporting Information). Compared with their counterparts, the mice also exhibited a significantly reduced immobility duration in both the TST and FST with AAV‐CMV‐Cre microinjection in HIP (Figure 2G) or IC (Figure 2H).

Major depression is generally associated with low bone mass and increased incidence of osteoporotic fractures.^[^ ^19^ ^]^ To investigate whether the knockdown of Mysm1 in MHb and HIP can alleviate bone loss, microcomputed tomographic (µCT) analysis was performed. µCT data of trabecular bones in the mouse femur revealed that mice in the stress group had a phenotype with obvious low bone mass (Figure 2I,K). The trabecular bone volume/tissue volume (BV/TV) ratio, trabecular thickness (Tb.Th) and trabecular number (Tb.N) were dramatically lower, accompanied by a higher trabecular separation (Tb.Sp), in mice exposed to chronic stress than in control mice, demonstrating osteoporosis in the stress group (Figure 2J,L). Surprisingly, AAV‐CMV‐Cre microinjection in MHb or in HIP for Mysm1 knockdown significantly attenuated the deficits in bone mass and structure (Figure 2I–L). These results demonstrated that Mysm1 knockdown alleviates not only the depression‐like behaviors but also osteoporosis in mice.

### 2.3 Astrocytic Mysm1 Knockdown Alleviated Depressive‐Like Behaviors in Mice

**Figure 3:**
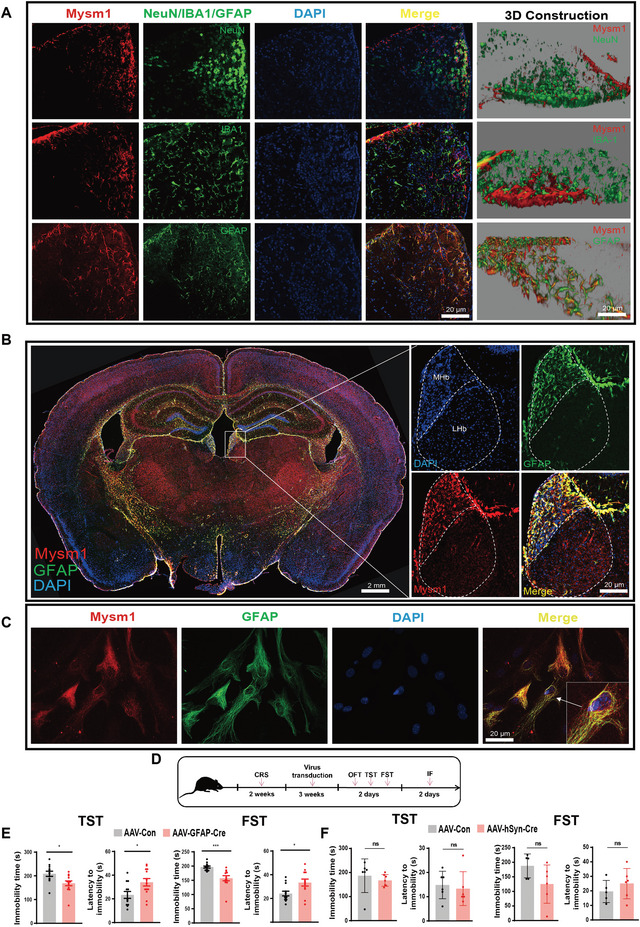
Astrocytic Mysm1 knockdown alleviates depressive‐like behaviors. A) Immunostaining of Mysm1 with the marker of neurons (NeuN), or microglia (IBA1), or astrocytes (GFAP) in MHb. 3D confocal images of the coimmunostaining were shown (left). Scale bar, 20 µm. B) Immunofluorescent staining of Mysm1 and GFAP in coronal brain sections from the CRS mouse model and higher‐magnification images of the MHb. Scale bar, 2 mm, 20 µm. C) Immunofluorescent imaging of Mysm1 in primary cultured astrocytes. D) Schematic diagram of virus injection and behavior tests after CRS model building. E) Knockdown of Mysm1 in astrocytes of CRS mice relieved depression‐like behaviors in the TST and FST. F) Knockdown of Mysm1 in neurons of CRS mice showed no significant relief of depression‐like behaviors in the TST and FST. Data represent the mean ± SEM; ns, not significant; *p < 0.05 and ***p < 0.001.

To explore which cell types expressed Mysm1, immunostaining was performed. Neuron (NeuN), microglia (IBA1), and astrocyte (GFAP) markers were costained with Mysm1. The costaining of GFAP and Mysm1 was prominent, while no evident costaining was observed in NeuN and IBA1 with Mysm1 (**Figure** **3**A). In addition, 3D rendering of confocal images showed that Mysm1 was expressed specifically in astrocytes (yellow), while there was no evident expression of Mysm1 in neurons and microglia (Figure 3A). Immunostaining of Mysm1 and NG2 for pro‐oligodendrocytes was carried out. As shown in Figure S5A (Supporting Information), no obvious costaining of Mysm1 and NG2 was found in MHb. Additionally, GFAP^+^ cells, CX3CR1^+^ cells, and NG2^+^ cells were sorted from brain of depressive mice and the expression of Mysm1 was examined. Mysm1 expression was detectable in GFAP^+^ cells but not CX3CR1^+^ cells and NG2^+^ cells (Figure S5B, Supporting Information). Next, we compared the colocalization of Mysm1 and GFAP in whole brain slices and the Mysm1 distribution in MHb by immunofluorescence. As expected, the brain regions with high expression of Mysm1 could be colocalized with GFAP in the MHb, HIP, and IC but hardly in the LHb (Figure 3B). Thereafter, primary astrocytes were isolated, and the expression of Mysm1 was detected by immunofluorescence. Staining data showed that Mysm1 was highly expressed in primary cultured astrocytes (Figure 3C).

To confirm that Mysm1 was located within astrocytes but not in neurons, we knocked down Mysm1 in either astrocytes or neurons. AAV expressing Cre recombinase under either the astrocytic GFAP promoter (AAV‐GFAP‐Cre) or the neuronal hSyn promoter (AAV‐hSyn‐Cre) was directly injected into the MHb of Mysm1^fl/fl^ mice with CRS exposure, and the behaviors were tested three weeks later (Figure 3D). Mysm1 knockdown in MHb was confirmed by qRT‐PCR (Figure S4C,D, Supporting Information). We found that mice with AAV‐GFAP‐Cre displayed a significantly reduced immobility duration in both the TST and FST (Figure 3E), while the AAV‐hSyn‐Cre group did not (Figure 3F). These results revealed that Mysm1 accumulation in astrocytes was involved in murine depressive‐like disorders.

### 2.4 Downregulation of Mysm1 Enhanced Mitochondrial Oxidative Phosphorylation and ATP Levels in Astrocytes and Improved Mitochondrial Structure, Alleviating Depression‐Like Behaviors in Mice

To investigate the molecular mechanisms by which astrocytic Mysm1 regulates depressive‐like behaviors, microarray, proteomics and metabolomics analysis were performed. The MHb and HIP from depressive mice and their control counterparts were collected for microarray analysis. The heatmap, GO enrichment and KEGG enrichment analysis data showed that many genes related to mitochondrial function were decreased in the MHb and HIP of depressive mice (Figure S6, Supporting Information). The ATP levels in the MHb and HIP were significantly lower in mice exposed to CRS than they were in control mice (Figure S7A,B, Supporting Information). Next, astrocytes transduced with AAV‐GFAP‐Cre to knockdown Mysm1 (AAV‐GFAP‐Cre) or control virus (AAV‐Con) were also collected for microarray analysis. Immunofluorescence and western blot data revealed that the expression of Mysm1 was significantly lower in AAV‐GFAP‐Cre cells than in AAV‐Con cells (Figure S8, Supporting Information). The pathways related to mitochondrial function were increased in cells with Mysm1 knockdown (Figure S9, Supporting Information).

**Figure 4:**
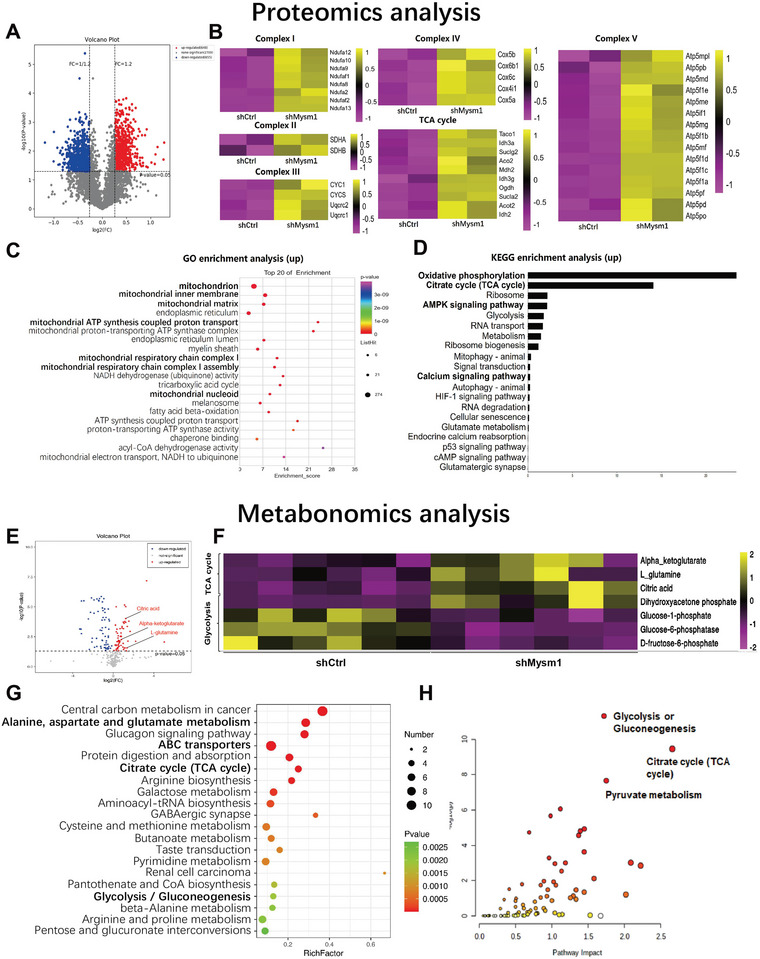
Downregulation of Mysm1 enhances mitochondrial oxidative phosphorylation in astrocytes. A) Volcano plot of differentially expressed genes between control (shCtrl) and Mysm1 knockdown (shMysm1) astrocytes. The X‐axis represents the fold change, while the Y‐axis indicates the significance of differential expression. The gray points indicate unigenes with no significant change (*p* < 0.05, false discovery rate (FDR) *q* < 0.05), while the redpoints and the blue points indicate up‐ and downregulated unigenes (*p* < 0.05, FDR *q* < 0.05), respectively. B) Heatmap showing hierarchical clustering of genes upregulated (yellow) or downregulated (pink) in astrocytes. C) Gene ontology enrichment gene number is defined as the number of target genes in each term. The rich factor is defined as the number of target genes divided by the number of all the genes in each term. The number of GO target genes, *p* value and rich factor are indicated in the scatterplot. D) KEGG pathway enrichment gene number is defined as the number of target genes in each pathway. KEGG pathways were calculated, and those with a *p* value < 0.05 were defined as significant. E) Volcano plot of differentially expressed metabolites between control (shCtrl) and Mysm1 knockdown (shMysm1) astrocytes. The X‐axis represents the fold change, while the Y‐axis indicates the significance of differential expression. The gray points indicate unigenes with no significant change (*p* < 0.05, false discovery rate (FDR) *q* < 0.05), while the redpoints and the blue points indicate up‐ and downregulated unigenes (*p* < 0.05, FDR *q* < 0.05), respectively. F) Heatmap showing hierarchical clustering of metabolites upregulated (yellow) or downregulated (pink) in astrocytes. G) KEGG pathway enrichment metabolite number is defined as the number of target metabolites in each pathway. KEGG pathways were calculated, and those with a *p* value < 0.05 were defined as significant. H) Analysis of Mysm1 downregulation in astrocytes using Metaboanalyst 5.0 Combined with Proteomic and Metabolomic Analysis.

To more comprehensively analyze the antidepressant mechanism caused by Mysm1 knockdown, proteomic and metabolomics analysis were performed to assess the differences between control (shCtrl) and Mysm1 knockdown (shMysm1) astrocytes. As shown in proteomic results, volcano plot analysis showed that there were 648 upregulated and 655 downregulated proteins in Mysm1 knockdown astrocytes (**Figure** **4**A). Heat map results revealed that many genes related to the mitochondrial respiratory chain and tricarboxylic acid (TCA) cycle were upregulated in Mysm1 knockdown astrocytes (Figure 4B). GO enrichment and KEGG enrichment analysis showed that the TCA cycle and AMPK pathway were upregulated in astrocytes with Mysm1 knockdown (Figure 4C,D). Metabolomics results showed that TCA cycle‐related metabolites such as citric acid, *α*‐ketoglutarate, and L‐glutamine were elevated in LV‐shMysm1‐transducted astrocytes as shown in volcano plot analysis and heat map results (Figure 4E,F). KEGG enrichment analysis revealed significant differences in TCA cycle, glycolysis and glutamate metabolism between astrocytes transducted with LV‐shMysm1and LV‐shCtrl (Figure 4G). Combined proteomics and metabolome analysis using Metaboanalyst 5.0 showed that the differences in astrocytes transducted with LV‐shMysm1 (shMysm1) and LV‐shCtrl (shCtrl) were mainly related to TCA cycle, glycolysis and pyruvate metabolism (Figure 4H). These results suggest that Mysm1 knockdown in astrocytes may upregulate TCA cycle process and mitochondrial oxidative phosphorylation levels to enhance mitochondrial function.

**Figure 5:**
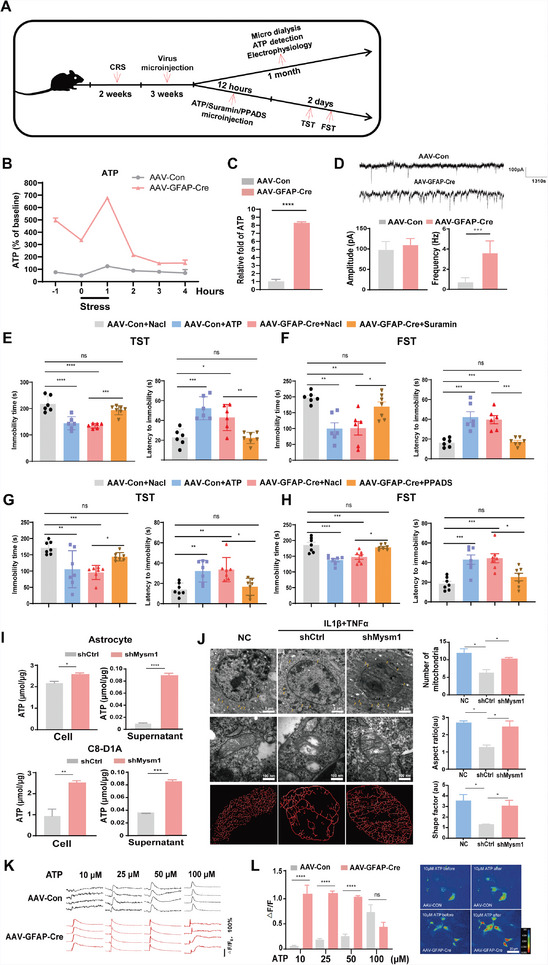
Mysm1 knockdown attenuates depression in an ATP‐dependent manner, improves mitochondrial structure in astrocytes and reduces calcium mobilization threshold. A) Schematic diagram of virus transduction, microdialysis, ATP detection, electrophysiological recording, microinjection of reagents and the behavior tests in CRS mice. B) Extracellular ATP levels in mouse hippocampus of CRS mice microinjected with AAV‐Con or AAV‐GFAP‐Cre virus. C) Relative ATP levels in the MHb from CRS mice microinjected with AAV‐Con (set as 1) or AAV‐GFAP‐Cre virus. D) Example traces of sEPSCs in MHb from CRS mice microinjected with AAV‐Con or AAV‐GFAP‐Cre virus. Amplitude and frequency were quantified. E–H) TST and FST results of Mysm1 downregulated and control depressive mice after injection of ATP and the non‐specific P2 receptor antagonist suramin or PPADS. I) ATP levels in primary cultured astrocytes and C8‐D1A cells and in their supernatant after transduced with the indicated viruses. J) Representative electron microscope images of mitochondria in astrocytes with indicative treatment and the number of mitochondria, aspect ratio and shape factor were quantified. Scale bar, 2 µm, 100 nm. K,L) Control and Mysm1 knockdown astrocytes respond to ATP. Four representative traces of [Ca^2+^]_i_ transients from four independent astrocyte lines loaded with the Ca^2+^ indicator Flou4/AM are shown. Scale bar, 20 µm. Data represent the mean ± SEM; ns, not significant; *p < 0.05, **p < 0.01, ***p < 0.001 and ****p < 0.0001.

Combined with the results that Mysm1 knockdown in astrocytes in vitro could produce more ATP, we explored whether Mysm1 knockdown in vivo could increase ATP in the interstitial space of the brain. We first subjected mice to chronic restraint stress and examined whether hippocampal and habenula ATP levels were decreased in depressive mice. The results showed that ATP levels in the hippocampus and habenula of depressive mice were significantly decreased (Figure S7A,B, Supporting Information). Next, AAV‐GFAP‐Cre or control virus (AAV‐Con) was injected into the CA1 region of the HIP in depressive Mysm1^fl/fl^ mice. After three weeks, the experiment was divided into two parts. A part of murine brains was used for in situ microdialysis, ATP testing, and electrophysiological recordings. Another part of mice was microinjected with ATP and the non‐specific P2 receptor antagonist suramin or pyridoxalphosphate‐6‐azophenyl‐2’,4’‐disulfonic acid (PPADS), and 12 h later, TST and FST behavioral assays were performed (**Figure** **5**A). As shown in Figure 5B, the ATP levels in the interstitial space of CRS mice with AAV‐GFAP‐Cre injection were significantly higher than those in the control mice. We also injected AAV‐GFAP‐Cre, or control virus, into the MHb of depressive Mysm1^fl/fl^ mice. The habenula were collected for ATP quantification. As expected, the ATP levels in the Mysm1 knockdown group were approximately eightfold higher than those in the control group (Figure 5C). In line with the high ATP level and improved behavioral data, an increased frequency of spontaneous excitatory postsynaptic currents (sEPSCs) in MHb was found in Mysm1 knockdown mice, indicating upregulation of neuronal activities (Figure 5D). Furthermore, TST and FST data showed that Mysm1 knockdown or ATP supplementation relieved depressive‐like behaviors in mice, suramin and PPADS reversed their antidepressant effects (Figure 5E‐H). These results suggest that Mysm1 exerts antidepressant effects by affecting ATP levels.

Given that energy production in astrocytes is largely dependent on mitochondrial oxidative metabolism in response to neuronal activity,^[^ ^20^ ^]^ we next tested whether Mysm1 knockdown in astrocytes could produce more ATP. We respectively cultured primary astrocytes and the astrocyte cell line C8‐D1A and transduced them with LV‐shMysm1 (shMysm1) or LV‐shCtrl (shCtrl) virus and ATP detection kit was used to test the ATP level in cells and supernatant. As expected, intracellular and extracellular ATP levels were significantly enhanced in the shMysm1 cells (Figure 5I).

To further explore the function of Mysm1 in mitochondrial biogenesis, we treated both shCtrl control and shMysm1 astrocytes with inflammatory factors (10 ng mL^−1^ IL1*β* plus 40 ng mL^−1^ TNF*α*). Electron microscopy was used to observe the morphological changes in the mitochondria of astrocytes. As expected, more mitochondria were observed in shMysm1 astrocytes (Figure 5J). Notably, Mysm1 knockdown enhanced the complexity of the tubular mitochondrial network in astrocytes (Figure 5J). In addition, ultrastructural defects in mitochondria, such as balloon‐like and disorganized cristae, were obvious in astrocytes treated with inflammatory factors, while the mitochondrial structures in shMysm1 astrocytes were relatively intact (Figure 5J). These results suggested that downregulation of Mysm1 enhances mitochondrial oxidative phosphorylation and ATP levels in astrocytes and improves mitochondrial structure.

Astrocytes can actively release ATP from vesicles or lysosomes in a calcium‐dependent manner.^[^ ^21^ ^]^ We found that intracellular calcium in shCtrl astrocytes did not increase significantly under 10 µM ATP treatment but gradually increased as the concentration of ATP used for stimulation increased (Figure 5K,L). However, the calcium signal in shMysm1 astrocytes could be stimulated by 10 µM ATP, and the calcium transient remained in a relatively activated state with an increase in ATP stimulation concentration (Figure 5K,L). These results suggested that the decrease of Mysm1 reduces the threshold of calcium mobilization and makes astrocytes easier to release more ATP.

### 2.5 Mysm1 Knockdown Activated the p53/AMPK Pathway

**Figure 6:**
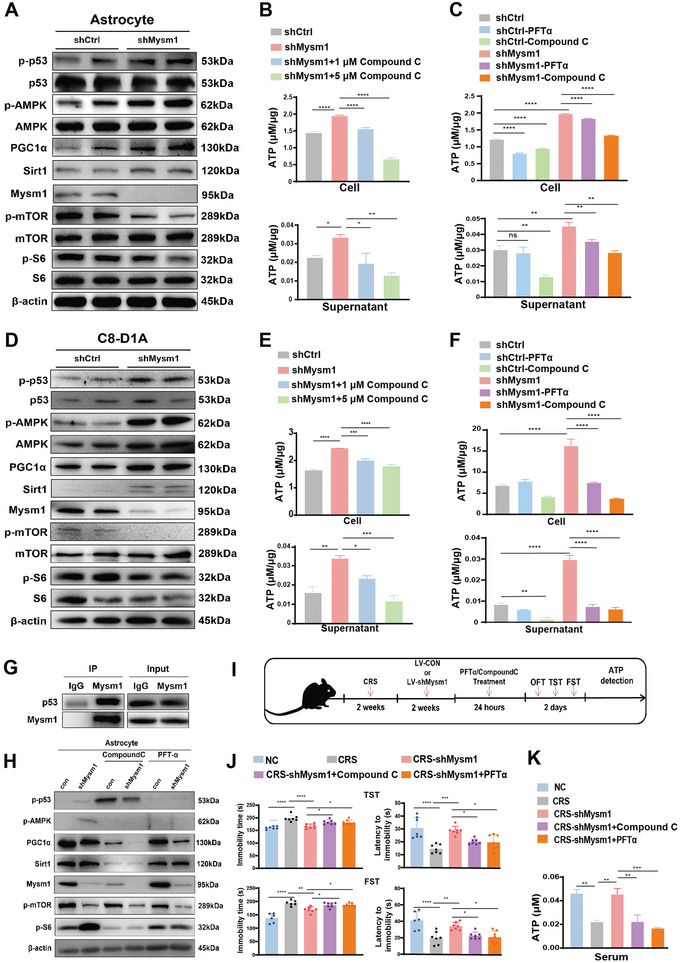
Mysm1 knockdown activates the p53/AMPK pathway and enhances ATP generation in astrocytes. A) Western blot of indicative proteins in primary astrocytes transduced with control (shCtrl) and Mysm1 knockdown (shMysm1) virus. B,C) ATP levels in astrocytes (top) and the supernatant (bottom) with the indicative treatment. D) Western blot of indicative proteins in C8‐D1A cells transduced with control (shCtrl) and Mysm1 knockdown (shMysm1) virus. E,F) ATP levels in C8‐D1A cells (top) and the supernatant (bottom) with the indicative treatment. G) The interaction between p53 and Mysm1 determined by Co‐immunoprecipitation (Co‐IP) in astrocytes. H) Western blot results in control and Mysm1 knockdown C8‐D1A cells stimulated with Compound C or PFT*α*. I) Schematic diagram of Compound C or PFT*α* treatment in Mysm1 knockdown CRS mice and the behavior tests. J) The TST and FST of mice with the indicative treatments in the MHb. K) ATP content in the serum of Compound C‐ and PFT*α*‐treated Mysm1 knockdown CRS mice and the control mice (NC). Data represent the mean ± SEM; ns, not significant; *p < 0.05, **p < 0.01, ***p < 0.001, and ****p < 0.0001.

As previously reported, Mysm1 deficiency could promote p53 pathway activation, thereafter, p53 could activate AMPK, which is involved in regulating the balance of energy metabolism.^[^ ^22^, ^23^ ^]^ We set out to verify whether Mysm1 knockdown can activate the p53/AMPK pathway in primary astrocytes and C8‐D1A cells and then to evaluate the ATP level. Western blot results showed that Mysm1 knockdown significantly enhanced the phosphorylation of p53 and AMPK (**Figure** **6**A,D).

The AMPK/PGC1*α*/Sirt1 signaling pathway plays an important regulatory role in mitochondrial biosynthesis.^[^ ^24^, ^25^ ^]^ Western blot assays confirmed that the expression of PGC1*α* and Sirt1 was increased in shMysm1 primary astrocytes and C8‐D1A astrocytes. In addition, mTOR and S6 phosphorylation, which are downstream of the AMPK pathway, were decreased (Figure 6A,D). To examine whether the p53/AMPK pathway governed ATP production, shCtrl or shMysm1 astrocytes or C8‐D1A cells were exposed to Compound C (AMPK inhibitor) or PFT*α* (p53 inhibitor). Intracellular and extracellular ATP contents were detected using an ATP assay kit. Compared with the shCtrl group, intracellular and extracellular ATP levels were significantly enhanced in both shMysm1 astrocytes (Figure 6B,C) and shMysm1 C8‐D1A cells (Figure 6E,F). Compound C and PFT*α* treatment reversed this effect (Figure 6C,F). Co‐immunoprecipitation (CO‐IP) results indicated that Mysm1 interacts with p53 (Figure 6G). Western blot analysis revealed that p53 phosphorylation was suppressed by PFT*α* and that AMPK activation was suppressed by both Compound C and PFT*α* (Figure 6H).

Next, we explored whether the inhibition of p53 and AMPK in vivo could reverse the antidepressant effect caused by Mysm1 knockdown. LV‐shMysm1 or LV‐shCtrl virus was injected into the MHb of CRS mice. Two weeks later, Compound C or PFT*α* was injected at the same site, and behavioral tests were performed 24 h after injection (Figure 6I). The TST and FST results showed that Compound C and PFT*α* significantly reversed the antidepressant effect caused by Mysm1 downregulation (Figure 6J). Interestingly, the ATP content of serum from Compound C‐ and PFT*α*‐treated Mysm1 knockdown mice was dramatically decreased compared to that of serum from Mysm1 knockdown mice (Figure 6K). Altogether, the data revealed that the knockdown of Mysm1 in astrocytes exerts an antidepressant effect by activating the p53/AMPK pathway.

### 2.6 Pharmacological Inhibition of Mysm1 Alleviated Depression‐Like Behaviors

**Figure 7:**
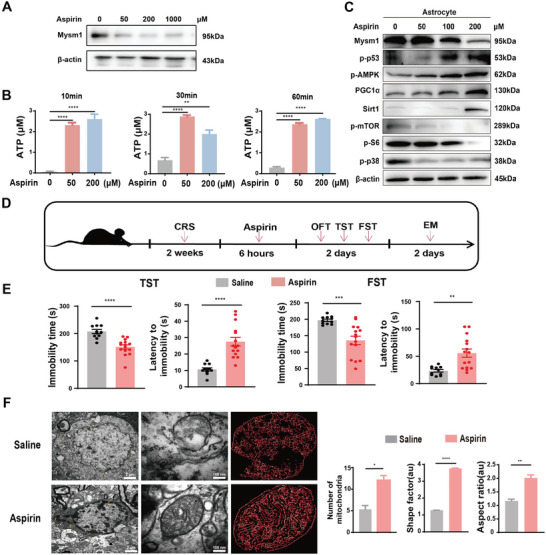
Inhibition of Mysm1 expression by aspirin alleviated murine depression‐like behaviors. A) Western blot of Mysm1 protein levels in cultured astrocytes treated with the indicative doses of aspirin. B) ATP levels in cultured astrocytes treated with the indicative doses of aspirin. C) Western blot of indicative proteins in cultured astrocytes treated with aspirin. D) Schematic diagram of aspirin treatment in CRS mice and the behavior tests. E) The TST and FST of CRS mice microinjected with saline or with aspirin at 12.5 µg µL^−1^. F) Representative electron microscope images of mitochondria in the MHb from CRS mice microinjected with saline or with aspirin and the number of mitochondria, aspect ratio and shape factor were quantified. Scale bar, 100 µm. Data represent the mean ± SEM; ns, not significant; *p < 0.05, **p < 0.01, ***p < 0.001, and ****p < 0.0001.

To investigate whether pharmacological reagents, which can inhibit Mysm1 expression, show antidepressant‐like effects, in vitro and in vivo assays were performed. Previous studies report that aspirin can reduce the expression of Mysm1 in the glomeruli of diabetic animals.^[^ ^26^ ^]^ Western blot data revealed that astrocytic Mysm1 protein levels were significantly decreased by aspirin treatment in a dose‐dependent manner (**Figure** **7**A). Surprisingly, ATP levels in astrocytes increased significantly even after 10 min of treatment with aspirin (Figure 7B). As shown in Figure 7C, aspirin treatment led to increased phosphorylation of p53 and AMPK and decreased phosphorylation of mTOR and S6 in astrocytes. Next, we examined the effect of aspirin on the behaviors of mice exposed to CRS. Aspirin was injected into the MHb of CRS mice, and 6 h later, behavioral tests and electron microscopy were performed (Figure 7D). Compared with mice injected with the control solvent, the immobility time in the FST and TST analysis was significantly reduced after aspirin injection (Figure 7E). The electron microscopy results revealed more mitochondria in the astrocytes in MHb slices treated with aspirin than in their control counterparts (Figure 7F). Under a high‐scale microscope, aspirin enhanced the complexity of the tubular mitochondrial network, and mitochondria in aspirin‐treated MHb cells had no obvious ultrastructural defects, such as balloon‐like and disorganized cristae, which could be quantified by a shape factor and aspect ratio (Figure 7F). Collectively, the data indicated that pharmacological drugs that suppress Mysm1 expression may be good candidates for depression therapy.

## 3 Discussion

**Figure 8:**
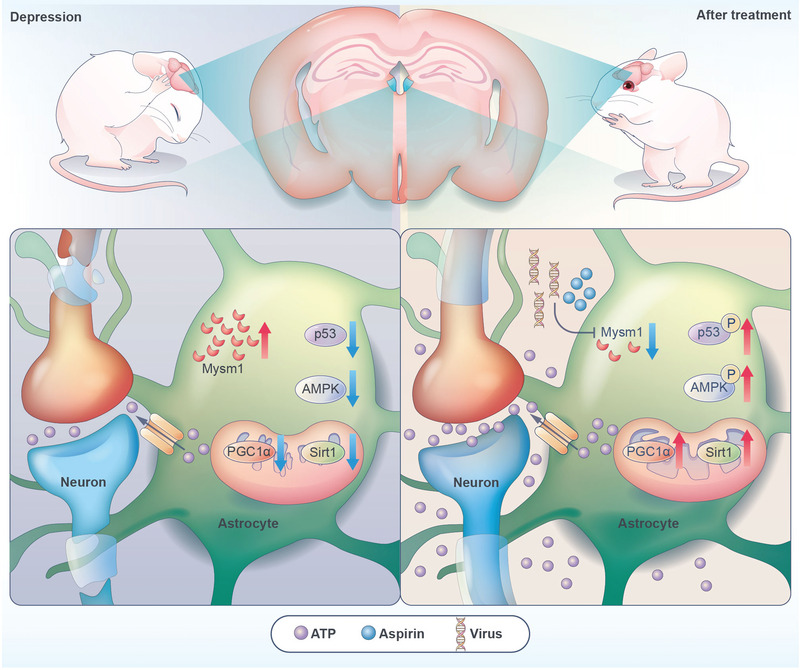
The model of the function of astrocytic Mysm1 in depression. The expression of Mysm1 was upregulated in astrocytes of depressive mice. Genetic and pharmacological inhibition of astrocytic Mysm1 alleviates depressive‐like disorders by promoting the activation of p53 and AMPK and then enhancing ATP production.

In the present study, we demonstrated that deubiquitinase Mysm1 was induced in the brain tissues from patients with major depression and mice with depressive‐like behaviors. The genetic silencing and pharmacological inhibition of astrocytic Mysm1 produced an antidepressant‐like effect by activating the p53/AMPK pathway and promoting ATP production (**Figure** **8**).

Mysm1 is an important regulator of diverse epigenetic signaling processes and the innate immune system. Mysm1 deficiency leads to abnormal hematopoiesis, hyperinflammation, enhanced viral clearance and tumor development.^[^ ^14^, ^27^, ^28^, ^29^, ^30^, ^31^ ^]^ This is the first study to demonstrate the special role of Mysm1 in brain function. Interestingly, Mysm1 was highly expressed in the hippocampus, internal capsule, frontal lobe, and temporal lobe brain sections from patients with severe depression and in the MHb, HIP, or IC of depressive mice. The knockdown of Mysm1 in the MHb, HIP, or IC alone is sufficient to alleviate depressive‐like behaviors in mice. Several studies have shown that the LHb, mPFC, and BLA are involved in regulating the depression phenotype.^[^ ^32^, ^33^, ^34^ ^]^ Our data demonstrated that no obvious expression of Mysm1 was observed in the LHb, mPFC, and BLA of mice exposed to CRS or LPS. The habenula can be divided into the LHb and MHb. The MHb is anatomically and histochemically different from the LHb.^[^ ^35^ ^]^ MHb is involved in regulating higher brain functions, including mood, reward, attention, and decision making.^[^ ^36^, ^37^, ^38^ ^]^ Studies have shown that MHb‐interpeduncular nucleus pathway and substance P play an important role in the pathogenesis of depression.^[^ ^39^, ^40^ ^]^ Our studies further confirm the differences between MHb and LHb and the important function of MHb in regulating depression. Costaining data for Mysm1 and GFAP revealed that Mysm1 is uniquely expressed in GFAP‐positive astrocytes. Astrocytic but not neural knockdown of Mysm1 relieves murine depressive‐like behaviors and surprisingly alleviates osteoporosis. Mysm1 was reported to accumulate in macrophages and regulate the immune response,^[^ ^41^ ^]^ However, Mysm1 is not detectable in microglia in CRS‐ or LPS‐induced depressive mice. Cell type‐specific expression of Mysm1 may indicate the specific function of Mysm1 in the brain.

Previous reports support the data in this study that showed decreased ATP levels in the habenula, HIP and several other brain regions in mice subjected to CRS or LPS.^[^ ^42^, ^43^ ^]^ A compromised level of ATP is implicated in depressive disorders, and ATP replenishment is sufficient to modulate depressive‐like behaviors in mice.^[^ ^43^ ^]^ Under normal physiological conditions, extracellular ATP is mainly released by astrocytes. Calhm2, a calcium homeostasis modulator family protein, governs astrocytic ATP release and modulates neural activity. The conditional astrocytic knockout of Calhm2 leads to reduced ATP concentrations and depression‐like behavior.^[^ ^10^ ^]^ Hippocampal CD39 contributes to chronic social defeat stress‐induced depression‐like behavior by hydrolyzing extracellular ATP.^[^ ^44^ ^]^ The astrocytic release of ATP is triggered by an increase in intracellular calcium concentration.^[^ ^45^ ^]^ These increases are predominantly elicited via the inositol 1,4,5‐trisphosphate (IP3) pathway.^[^ ^46^ ^]^ Itpr2‐deficient mice exhibited depressive‐like behaviors.^[^ ^43^ ^]^ In these models, treatment with ATP reverses depressive behaviors. Here, we found that astrocytic Mysm1 knockdown in MHb cells promoted ATP production and induced antidepressant effects, which further confirmed the important role of astrocytes in energy modulation. Normal mitochondrial function is essential for ATP production.^[^ ^47^ ^]^ Mitochondrial dysfunction leads to depressive‐like behaviors.^[^ ^47^ ^]^ Under IL1*β* and TNF*α* stimulation, ultrastructural defects of mitochondria, such as balloon‐like and disorganized cristae, were obvious in astrocytes, while the mitochondrial structures in Mysm1 knockdown astrocytes were relatively intact. The expression of PGC1*α* and Sirt1 increased consistently with decreased Mysm1. Our results further verified that increasing mitochondrial biogenesis and ATP production in astrocytes is an ideal strategy for alleviating depressive disorders.

AMPK is an important factor in maintaining energy homeostasis, and its core function is closely related to ATP levels.^[^ ^48^ ^]^ Our microarray, proteomics analysis and metabolomics analysis data showed a significant increase in the citrate cycle and AMPK pathway, which are essential for ATP production. During activation, AMPK activates ATP‐generating pathways and inhibits ATP‐consuming pathways.^[^ ^49^ ^]^ With Mysm1 knockdown, AMPK was dramatically activated, and mTOR signaling was suppressed. When Compound C, which opposes the AMPK pathway, was added to the culture system, or injected into the MHb, the ATP levels in astrocytes were decreased, and the antidepressant effect of Mysm1 suppression was reversed. These data further confirmed the role of Mysm1 in the regulation of the AMPK pathway in astrocytes.

Mysm1 is essential for hematopoiesis and lymphogenesis through the epigenetic regulation of the expression of cell type‐specific transcription factors.^[^ ^14^, ^30^, ^50^, ^51^ ^]^ In bone marrow‐derived macrophages or peripheral blood monocytes, Mysm1 can interact with STING, TRAF3, and TRAF6, cleave their ubiquitination, and attenuate the related pathway.^[^ ^11^, ^12^ ^]^ In macrophages, Mysm1 deficiency leads to p38 MAPK activation.^[^ ^15^ ^]^ However, Mysm1 did not regulate the p38 MAPK pathway in astrocytes (data not shown). Several studies have reported that Mysm1 can interact with the p53 axis in the process of tissue development.^[^ ^27^, ^52^ ^]^ p53 has been shown to activate the AMPK pathway and suppress the mTOR pathway in glioma cells.^[^ ^53^ ^]^ Interestingly, the increased phosphorylation of p53 was observed with Mysm1 knockdown in astrocytes. The inhibition of p53 function using PFT*α* not only suppressed p53 activation but also AMPK activation and reduced ATP production in both primary astrocytes and the astrocyte cell Line C8‐D1A. Consistently, microinjection of PFT*α* in the MHb attenuated the antidepressant effect of Mysm1 downregulation. Guo et al. reported that p53 can be stabilized and then activated by K63‐linked ubiquitination.^[^ ^54^ ^]^ Whether Mysm1 can affect the ubiquitination of p53 and regulate p53 activation in astrocytes requires further investigation.

Our findings demonstrate that repressing Mysm1 expression could relieve depression‐related behaviors. A previous report in support of our data demonstrated that aspirin was able to suppress Mysm1 expression in diabetic animals.^[^ ^26^ ^]^ Preclinical, pharmacoepidemiologic and pilot clinical trial data have suggested that aspirin has therapeutic potential for depression due to its anti‐inflammatory properties.^[^ ^55^ ^]^ Here, we found that microinjection of aspirin into the MHb alleviated depressive‐like behavior and restored mitochondrial structure in vivo in mice. Astrocytes produced and released more ATP following aspirin treatment. Henry et al. reported that aspirin suppressed breast cancer cell growth by activating AMPK and inhibiting mTORC1 signaling.^[^ ^56^ ^]^ In astrocytes treated with aspirin, decreased Mysm1, the activation of p53 and AMPK, and the suppression of mTORC1 signaling were observed. Accordingly, higher ATP production and ATP release in astrocytes were induced by aspirin. Metformin was reported to ameliorate stress‐induced depression‐like behaviors by activating AMPK and cAMP‐response element binding protein.^[^ ^57^ ^]^ In the future, it would be interesting to examine other antidepressant reagents for their ability to relieve depression by suppressing Mysm1 expression in astrocytes. Mysm1 mutations in both mice and patients showed similar developmental aberrations and functional disorders.^[^ ^14^, ^29^ ^]^ Le Guen et al. reported that a patient with a homozygous Mysm1 mutation presented with a complete lack of B lymphocytes, defective hematopoiesis, and development abnormalities. The patient recovered a normal immunohematologic phenotype because of a spontaneous genetic reversion of the Mysm1 mutation in a hematopoietic stem cell.^[^ ^29^ ^]^ We found that Mysm1 expression was significantly increased in brains from both aged patients with major depression diseases and mice with depressive‐like behaviors. Hopefully, reducing astrocytic Mysm1 expression to alleviate depression in mice will also be an ideal strategy in humans suffering from depressive disorders.

In summary, this study revealed that elevated Mysm1 in astrocytes aggravated depressive‐like behavior in mice induced by chronic stress and LPS. Suppression of Mysm1 expression could relieve depressive disorders by promoting ATP production. These results highlight that aberrant astrocytic Mysm1 functions as a potential molecular mechanism underlying MDD pathophysiology, and suggest that astrocytic Mysm1 could serve as a potential target for new therapeutic interventions against depressive disorders.

## 4 Experimental Section

### Autopsy Samples of Human Brain

The brain tissues of four elderly patients with major depression and four non‐depressive elderly patients were provided by National Human Brain Bank for Development and Function, Chinese Academy of Medical Sciences and Peking Union Medical College, Beijing, China. This study was supported by the Institute of Basic Medical Sciences, Chinese Academy of Medical Sciences, Neuroscience Center, and the China Human Brain Banking Consortium (009‐2014).

### Animals

All animal experiments were conducted according to the Guide for the Care and Use of Laboratory Animals by the Administrative Panel on Laboratory Animal Care at the Institute of Basic Medical Sciences (Beijing, China) (iacuc‐dwzx‐2021‐758). The floxed Mysm1 mouse strain (Mysm1^fl/fl^) were a kind gift from Dr. Si‐Yi Chen in University of Southern California. Mysm1^fl/fl^ mice (8–12 weeks of age) were used for establishing the LPS/CRS‐treated depressive‐like models, virus microinjection in the behavior experiments and molecular biological detection. All male or female C57BL/6N mice (8‐12 weeks of age) were purchased from Charles River (Beijing, China). Four to five mice were housed in a plastic cage (300 × 120 × 170 mm) and maintained in a circadian cycle of 12 h light (8:00 a.m.–8:00 p.m.) and 12 h dark (8:00 p.m.–8:00 a.m.) with an adequate food and water supply.

### Cell Cultures

For primary astrocytes isolation and culture, primary astrocytes were dissected from newborn Mysm1^fl/fl^ or C57BL/6N mice. The brain cortex and hippocampus were dissected, and the meninges were removed under a microscope. Then, the tissue was dissociated by enzymatic digestion, and the isolated astrocytes were cultured in astrocytic medium (Dulbecco's Modified Eagle's Medium/Nutrient mixture F‐12 Ham) (DMEM/F12) (1:1) (Sigma, D8437) with 10% fetal bovine serum (FBS; ExCell Bio, 12B013) and antibiotics (100 U mL^−1^ penicillin and 100 µg mL^−1^ streptomycin, Gibco) in a dish plated with poly‐D‐lysine (Sigma, P6407) and cultured in a 5% CO_2_ incubator at 37 °C. Astrocytes were grown for ≈7 days until the cultures reached the desired density, with medium changes every 2 days. Trypsin‐EDTA solution (0.25%, Sigma) was used to dissociate primary astrocytes. Cells from passage 2–3 were used for the experiments.

For C8‐D1A astrocytes, cells were cultured in Dulbecco's modified Eagle's medium (DMEM; Gibco, Thermo Fisher Scientific, D6429) with high glucose and supplemented with 10% FBS and antibiotics at 37 °C in humidified 5% CO_2_ incubator. Trypsin‐EDTA solution (0.25%, Sigma) was used to dissociate C8‐D1A cells. Cells from passage 2–15 were used for the experiments.

### Drug Administration

ATP powder (Sigma, A2383) was dissolved in normal saline at a final concentration of 50 mg mL^−1^. The concentration of 10, 25, 50, and 100 µM was used for subsequent cell stimulation. For behavioral experiments, 50 µM ATP was delivered to MHb of depressive mice by automatic sampler (Hamilton 701rn) for subsequent behavioral analysis.

Calcium ion fluorescent indicator Fluo‐4, AM powder (Invitrogen, 2071565) was dissolved in an appropriate amount of dimethyl sulfoxide (DMSO) to make a 1 mm stock solution; Fluo‐4, AM stock solution was diluted with HBSS to 2 µm working solution and used for subsequent astrocyte calcium signal detection.

10 ng mL^−1^ IL1*β* (PeproTech, 211‐11B) and 40 ng mL^−1^ TNF*α* (PeproTech, 315‐01A) were used to stimulate primary astrocytes for 24 h and collected for analysis.

5 µM PFT*α* (Abmole, M2036) and 1 or 5 µm Compound C (Abmole, M2238) were used to stimulate primary and C8‐D1A astrocytes for 24 h and collected for analysis. PFT*α* (0.4 µg µL^−1^) or vehicle (10% DMSO in saline) was injected into the MHb (AP, −1.40 mm from bregma; ML, ± 0.25 mm; DV, −2.60 mm from the brain surface) at a volume of 1 µL per mice. Compound C was dissolved in DMSO (3 mg mL^−1^) and was injected into the MHb (AP, −1.40 mm from bregma; ML, ± 0.25 mm; DV, −2.60 mm from the brain surface) at a volume of 1 µL per mice.

Suramin (5 µM, Sigma) and PPADS (1 nmol µL^−1^, Tocris) were injected into mice MHb (AP, −1.40 mm from bregma; ML, ± 0.25 mm; DV, −2.60 mm from the brain surface) at a volume of 1 µL per mice.

### Immunohistochemistry

Mouse brains were dissected and fixed with 4% paraformaldehyde in PBS for three days at room temperature. Then, the brains were dehydrated with a sucrose gradient (10%, 20%, and 30%) in PBS. The 40‐µm coronal brain slices were sectioned with a Leica CM3050S (Leica). Brain slices were permeabilized in PBS twice and incubated in blocking buffer (PBS containing 0.4% Triton X‐100, 2% goat serum, and 1% bovine serum albumin) for 1 h at room temperature. The primary antibodies were GFAP (Merckmillipore, MAB360), NeuN (Abcam, ab104225), Mysm1 (Abcam, ab193081), and IBA1 (Abcam, ab178847), and the slices were incubated with fluorescence‐conjugated secondary antibodies (Jackson Immunoresearch) for 1 h at room temperature. Images of stained brain slices were acquired using a confocal microscope (Leica). The region of interest was divided into 10 equal laminar blocks representing different areas of target tissues. Positive cells were counted and analyzed by using ImageJ software. Human brain paraffin section on a saline‐coated slide was boiled in citrate buffer (pH = 6, ZLI9065, ZSGB‐BIO) for 5 min for deparaffinization and rehydration.^[^ ^58^ ^]^ Next, incubated with appropriately diluted Mysm1 (1:1000, Abcam) antibody for 1 h at room temperature. After washing with Tris‐buffered saline containing 0.1% Tween‐20 (TBS‐T), the specimens were sequentially incubated for 20 min with biotinylated anti‐rabbit immunoglobulins and peroxidase‐labelled streptavidin. Staining was performed after 10 min incubation with a freshly prepared substrate‐chromogen solution containing 3% 3‐amino‐9‐ethylcarbazole and hydrogen peroxide. Finally, the slides were lightly counterstained with hematoxylin, washed with water, and then mounted.

### Quantitative RT–PCR

Total RNA from cells and tissues was extracted and isolated by TRIzol (Invitrogen). Total RNA was extracted by chloroform extraction and isopropanol precipitation according to the manufacturer's recommendations. Reverse transcription was performed using ReverTra Ace qPCR RT Master Mix (Toyobo, 037400). Quantitative PCR was performed with 2 × T5 Fast qPCR Mix (SYBR) (TSINGKE, TSE202). Each amplification cycle consisted of an initial step at 95 °C (5 min), followed by 40 cycles of denaturation at 95 °C for 15 s, annealing at 60 °C for 1 min, and extension at 72 °C for 30 s. GAPDH or *β*‐actin was used as an internal control.

### Western Blot

Cells and tissues were homogenized in lysis buffer (RIPA) on ice for 40 min and subsequently centrifuged at 12 000 rpm for 5 min at 4 °C. Then, the supernatants were transferred to a clean 1.5 mL tube. Samples containing 30 µg of protein were separated using 12% SDS–PAGE gels. Proteins were transferred onto PVDF (polyvinylidene difluoride) membranes in cold buffer (25 mm Tris Base, 192 mm glycine) by electrotransfer for 1.5 h, which were incubated in TBS buffer containing 5% milk for 1 h at 22–24 °C. The membrane was probed at 4 °C overnight with the following primary antibodies: Rabbit anti‐Mysm1 (1:1000, Abcam), rabbit anti‐PGC1*α* (1:1000, Cell Signaling Technology), rabbit anti‐p‐p53 (1:1000, Cell Signaling Technology), rabbit anti‐p53 (1:1000, Cell Signaling Technology), rabbit anti‐p‐AMPK (1:1000, Cell Signaling Technology), rabbit anti‐AMPK (1:1000, Cell Signaling Technology), rabbit anti‐Sirt1 (1:1000, Cell Signaling Technology), rabbit anti‐p‐mTOR (1:1000, Cell Signaling Technology), rabbit anti‐mTOR(1:1000, Cell Signaling Technology), rabbit anti‐p‐S6 (1:1000, Cell Signaling Technology), mouse anti‐S6 (1:1000, Cell Signaling Technology), rabbit anti‐GAPDH (1:1000, Abclonal), mouse anti‐*β*‐actin (1:1000, APPLYGEN or Cell Signaling Technology). After 3 washes, the membranes were incubated with secondary antibodies in TBS buffer for 1 h at 22–24 °C. Enhanced chemiluminescence (APPLYGEN) was used to visualize the immunoreactive bands. The secondary antibodies included HRP goat anti‐rabbit IgG (1:2000, Abclonal) and HRP goat anti‐mouse IgG (1:2000, APPLYGEN).

### Co‐Immunoprecipitation

Astrocytes were lysed with cold IP‐lysis buffer (20 mm Tris‐HCl ([pH 7.4)], 0.15 m NaCl, 1 mm EDTA, 1 mm EGTA, 1% Triton X‐100 with protease inhibitors cocktail (CST) and 500 mm Dithiothreitol (DTT, CST) for 3 h and centrifuged at 12 000 rounds per minute (rpm) for 15 min at 4 °C. Supernatant of whole cell lysates were transferred into three new tubes. The protein was incubated with Anti‐Mysm1(Abcam) or IgG (for control, CST) for overnight at 4 °C, then mixed with Agarose Aagarose A/G for 2 h. The precipitants were washed three times with IP‐lysis buffer, dissolved in sample loading buffer and heat denaturing with 4 × laemmli sample buffer (Omiget, china) and subsequently expressions of the target proteins were determined by western blot.

### Cell Isolation by Fluorescence‐Activated Cell Sorting (FACS)

Hippocampus and Cerebral Cortex from LPS‐induced mice were dissociated by using Adult Brain Dissociation Kit (130‐107‐677, Miltenyi Biotec) according to the manufacturer's instructions. Primary antibody against pro‐pro‐oligodendrocytes (NG2, ab275024, Abcam) was added and cells were incubated for 30 min on ice in the dark. After washing three times in staining buffer (containing phosphate‐buffered saline, pH 7.2, 0.5% fetal bovine serum and 2 mm EDTA), cells were staining with second antibody (PE Donkey anti‐rabbit IgG, 406421, Biolegend). After washing, antibodies against astrocytes (Rabbit Anti‐ GFAP/AF488, bs‐0199R‐AF488, Bioss) and microglia cells (APC anti‐mouse CX3CR1, 149007, Biolegend) were added and incubated. Cells were resuspended in 500 µL staining buffer and sorted by using a FACS Aria II sorter (BD). Sorted cells were pelleted and subjected to RNA extraction with MolPure Cell RNA Kit (Yeason Biotech, 19231ES50) according to the manufacturer's instructions.

### Depression Model and Behavior Assay

For Chronic restraint stress depression model, mice were placed in 50 mL conical tubes with holes for fresh air for 2–3 h per day for 14 consecutive days.^[^ ^18^ ^]^ For LPS‐induced depression model, LPS (Sigma, L‐2880) was dissolved in aseptic 0.9% saline. Mice were exposed to 10 days of LPS (0.5 mg kg^−1^ per day, intraperitoneal (i.p.)) between 09:00 a.m. and 10:00 a.m.^[^ ^32^ ^]^ The behavior tests were performed 24 h after CRS and LPS modeling.

In open field test, mice were placed in the center of a rectangular chamber (40 × 40 × 30 cm) in a silent room with dim light for 5 min.^[^ ^43^ ^]^ An automated video camera positioned above the chamber was used to track and analyze the digitized image of the path (Any‐maze, Stoelting). This test was performed in the morning (08:00–11:00 a.m.).

Tail suspension test. Mice were suspended on a frame (50 cm above the floor) for 6 min.^[^ ^10^ ^]^ The latency to immobility in the first period and the cumulative immobility time during the final 4 min were recorded by an investigator blinded to the groups. This test was performed in the afternoon (2:00–6:00 p.m.).

In forced swimming test, mice were placed in a cylinder (12 cm diameter, 30 cm height) of water (21–25 °C) and swam for 6 min under normal light.^[^ ^32^ ^]^ The latency to immobility in the first period and the cumulative immobility time during the final 4 min were recorded by an observer blinded to the groups.

For coat score assay, the physical state of the fur was observed daily by an investigator blinded to the groups, and the total coat score was measured as the sum of the score of seven different body parts: head, neck, dorsal coat, ventral coat, tail, forepaws and hindpaws.^[^ ^43^ ^]^ For each of the seven areas, a score of 1 was given for a well‐groomed coat, and a score of 0 was given for a messy coat.

For behavioral test, all mice were tested for three independent times. The behavioral tests were performed during the light cycle (10:00 to 17:00). Before tested, mice were put in the testing room for sixty‐minute adapting.

### Stereotaxic Surgery and Virus Injection

Mice (P48‐60) were anesthetized using 2,2,2‐tribromoethanol (Sigma, 240 mg kg^−1^ of body weight). Animals were placed on a stereotactic frame (RWD). Adeno‐associated viruses (AAV‐Con, AAV‐CMV‐Cre, AAV‐GFAP‐Cre, AAV‐hSyn‐Cre) and lentiviruses (LV‐Ctrl, LV‐shMysm1) were purchased from Hanbio Biotechnology Co., Ltd. Viruses were stereotactically injected into the MHb (AP, −1.40 mm from bregma; ML, ± 0.25 mm; DV, −2.60 mm from the brain surface) or HIP (AP, −1.70 mm from bregma; ML, ± 1.25 mm; DV, −1.75 mm from the brain surface) or IC (AP, −1.70 mm from bregma; ML, ± 2.50 mm; DV, −4.00 mm from the brain surface) bilaterally with a pressure microinjector (Hamilton 701RN) at a slow rate of 0.1 µL min^−1^. The injection needle was withdrawn 10 min after the infusion. Behavioral tests were performed 3 weeks after AAV injections.

### Microdialysis

Mice were anesthetized with isoflurane and fixed on a brain stereotaxic frame. The head skin was disinfected with 75% alcohol to expose the skull surface. A microdialysis guide cannula (CMA/10, CMA, Stockholm, Sweden) was implanted into the left hippocampus (AP, −1.94 mm from bregma; ML, 1.0 mm; DV, 1.2 mm from the brain surface). Dental cement (glass cement) was used to hold the probe sleeve in place. The next day, the inner core of the probe sleeve was removed and perfused with compound sodium chloride injection at a rate of 1 µL per minute. The samples were stored at −80 °C.

### Calcium Imaging

Cells were cultured on glass‐bottom dishes (NEST, 801001, Shanghai, China) and loaded with 5 ng µL^−1^ Fluo‐4 AM (Thermo Fisher Scientific, Waltham, MA, USA) at 37 °C for 30 min while cells reached 60–70% confluence. Then, the medium was washed three times with 1 × HBSS and incubated with culture medium at 37 °C for 30 min. The dish was affixed on an automatic inverted confocal microscope (Nikon Eclipse Ti‐E) equipped with a Perkin Elmer Ultra View rotating disc scanner unit, and the excitation was performed at 488 nm. The buffer was replaced with experimental buffer containing 0, 25, 50, or 100 µM ATP. [Ca^2+^]i transients were expressed in the form of ΔF(t)/F_0_, where F0 is the baseline fluorescence of a given region of interest and ΔF is the difference between the current level of fluorescence F(t) and F0.

### Proteome Analysis

Mysm1 knockdown or control C8‐D1A cells were used per biological replicate. Frozen samples were transferred into low protein binding tubes (1.5 mL Eppendorf) and lysed with 300 µL lysis buffer supplemented with 1 mm PMSF. Then, the samples were further lysed with sonication. SDS was added to a concentration of 2% for efficient solubilization before using a modified FASP protocol.^[^ ^59^ ^]^ Proteomic measurements were performed on a Q Exactive mass spectrometer (Thermo Fisher) coupled to an Ultimate 3000 liquid phase system (Thermo Fisher).

### Metabolome Analysis

For sample preparation, using ultrasonic homogenizer to breaking up the astrocytes for 6 min at 500 w. All of the mixtures of each sample were transferred to 1.5 mL Eppendorf tubes, L‐2chlorophenylalanine (0.3 mg mL^−1^) dissolved in methanol as internal standard, then extracted by ultrasonication for 20 min in ice‐water bath. The extract was centrifuged at 4 °C (13 000 rpm) for 10 min. Supernatant in a glass vial was dried in a freeze concentration centrifugal dryer. QC sample was prepared by mixing aliquot of the all samples to be a pooled sample. An aliquot of the supernatant was transferred to a glass sampling vial for vacuum‐dry at room temperature. 15 mg mL^−1^ methoxylamine hydrochloride in pyridine was subsequently added. The resultant mixture was vortexed vigorously for 2 min and incubated at 37 °C for 90 min. BSTFA (with 1% TMCS) and *n*‐hexane were added into the mixture, which was vortexed vigorously for 2 min and then derivatized at 70 °C for 60 min. The samples were placed at ambient temperature for 30 min before GC‐MS analysis.

The gas chromatography‐mass spectrometry (GC‐MS) protocol was similar as previously described in ref.^[^ ^60^ ^]^ The derivatived samples were analyzed on an Agilent 7890B gas chromatography system coupled to an Agilent 5977A MSD system (Agilent Technologies Inc., CA, USA). A DB‐5MS fused‐silica capillary column (30 m × 0.25 mm × 0.25 µm, Agilent J & W Scientific, Folsom, CA, USA) was utilized to separate the derivatives. Helium (>99.999%) was used as the carrier gas at a constant flow rate of 1 mL min^−1^ through the column. The injector temperature was maintained at 260 °C. The initial oven temperature was 60 °C held at 60 °C for 0.5 min, ramped to 125 °C at a rate of 8 °C min^−1^, to 210 °C at a rate of 5 °C min^−1^, to 270 °C at a rate of 10 °C min^−1^, to 305 °C at a rate of 20 °C min^−1^, and finally held at 305 °C for 5 min. The temperature of MS quadrupole and ion source (electron impact) was set to 150 and 230 °C, respectively. The collision energy was 70 eV. Mass spectrometric data was acquired in a full‐scan mode (m/z 50–500).

The obtained GC/MS raw data in .D format were transferred to .abf format via software Analysis Base File Converter for quick retrieval of data. Then, data were imported into software MS‐DIAL, which performs peak detection, peak identification, MS2Dec deconvolution, characterization, peak alignment, wave filtering, and missing value interpolation. The matrix was imported in R to carry out Principle Component Analysis (PCA) to observe the overall distribution among the samples and the stability of the whole analysis process. Orthogonal Partial Least‐Squares‐Discriminant Analysis (OPLS‐DA) and Partial Least‐Squares‐Discriminant Analysis (PLS‐DA) were utilized to distinguish the metabolites that differ between groups. To prevent overfitting, 7‐fold cross‐validation and 200 Response Permutation Testing (RPT) were used to evaluate the quality of the model.

### High‐Throughput RNA Sequencing (RNA‐Seq)

Total RNA was extracted from the cells and mouse habenula using TRIzol reagent (Invitrogen, 10296010) according to the manufacturer's protocol. RNA purity and quantification were evaluated using a NanoDrop 2000 spectrophotometer (Thermo Scientific, USA). RNA integrity was assessed using the Agilent 2100 Bioanalyzer (Agilent Technologies, Santa Clara, CA, USA). Then, the libraries were constructed using the TruSeq Stranded mRNA LT Sample Prep Kit (Illumina, San Diego, CA, USA) according to the manufacturer's instructions. Transcriptome sequencing and analysis were conducted by OE Biotech Co., Ltd. (Shanghai, China). The libraries were sequenced on an Illumina HiSeq X Ten platform, and 150 bp paired‐end reads were generated. Raw data (raw reads) in fastq format were first processed using Trimmomatic, and the low‐quality reads were removed to obtain clean reads.

### Transmission Electron Microscopy (TEM)

For TEM, Cells or MHb slices were fixed in 2.5% glutaraldehyde in 0.1 M sodium cacodylate buffer and postfixed in 2% osmium tetroxide in 0.1 M sodium cacodylate buffer with 0.3% potassium ferrocyanide, stained with 4% aqueous uranyl acetate, dehydrated, infiltrated and embedded in epoxy resin. Ultrathin sections (70 nm) were cut by a LEICA EM UC6 microtome and imaged using an electron microscope (Hitachi H‐7650).

### Analysis of Mitochondrial Morphology and Shape

Electron microscope images were analyzed by ImageJ. A shape factor consisting of P^2^/4*π*A (p, perimeter and A, area) was applied to mitochondria. An aspect ratio consisting of d_max_/d_min_ (d, dome) was applied to mitochondria.^[^ ^61^ ^]^

### Slice Preparation

Male mice (aged 8 weeks) were anesthetized with pentobarbital and decapitated, and the brains were quickly removed and placed in ice‐cold oxygenated modified ACSF containing 195 mm sucrose, 1.3 mm NaH_2_PO_4_, 0.2 mm CaCl2, 26 mm NaHCO_3_, 12 mm MgSO_4_, 2 mm KCl, and 10 mm glucose. Coronal MHb slices (300 µm) were prepared using a VT‐1200S vibratome (Leica, Germany), subsequently transferred to a storage chamber containing normal ACSF (10 mm glucose, 3.0 mm KCl, 126 mm NaCl, 1.25 mm NaH_2_PO_4_, 2.0 mm CaCl_2_, 1.0 mm MgSO_4_, and 26 mm NaHCO_3_), incubated for 30 min at 34 °C for recovery and subsequently incubated at room temperature (25 ± 1 °C) for an additional 2–7 h. All solutions were saturated with 95% O_2_/5% CO_2_ (vol/vol).

### Electrophysiological Recordings

The slices were placed in a recording chamber that was perfused with artificial cerebrospinal fluid (2 mL min^−1^) at 32–34°C. Whole‐cell patch‐clamp recordings were obtained under IR‐DIC visualization (Zeiss, Axioskop 2). The pipettes were pulled with a micropipette puller (P‐97, Sutter instrument) with a resistance of 3–5 MΩ. After establishing the whole‐cell configuration, neurons were held at −70 mV to record sEPSCs. To record sEPSCs, pipettes were filled with an intracellular solution containing: 130 mm potassium gluconate, 20 mm KCl, 10 mm HEPES buffer, 4 mm Mg‐ATP, 0.3 mm Na‐GTP, 10 mm disodium phosphocreatine and 0.2 mm EGTA, at 290 mOsm pH 7.2, adjusted with KOH. When sEPSCs were recording, the GABAA receptors were blocked with 20 µm bicucullinemethiodide (BMI). The data were recorded with a Multiclamp 700B (Molecular Devices), digitized at 10 kHz, filtered at 2 kHz, collected when the series resistance fluctuated within 20% of the initial values and analyzed using Clampfit 10.2 software (v.10.6.2.2., Molecular Devices). A fixed length of traces (5 min) was analyzed for the frequency and amplitude distributions of sEPSCs. In all experiments, series resistance was controlled below 50 MΩ and not compensated. Cells were rejected if series resistance fluctuated more than 50 MΩ or 25% of initial values.

### Microcomputed Tomographic (µCT) Analysis

Femurs (one per mouse, *n* = 4) were fixed in phosphate‐buffered formalin for 48 h and further preserved in 70% ethanol. Specimens were scanned at 10micron isotropic resolution using a Skyscan 1276 (Bruker, Billerica, MA). In briefly, scans were performed at a 20‐µm resolution in all three spatial dimensions. The mineralized tissues were differentially segmented by a global thresholding procedure.^[^ ^62^ ^]^ The following morphometric parameters were determined by using a direct 3D approach: mineralized volume fraction (BV/TV) (%), trabecular thickness (Tb.Th.), trabecular number (Tb.N.) and trabecular separation (Tb.Sp.).

### Statistical Analysis

Data are presented as the mean ± SEM. Two‐way ANOVA was used to analyze all behavioral tests between and among the treatment groups. In anatomical and biochemical studies, one‐way or two‐way ANOVA was used to compare multiple groups. A Bonferroni post hoc analysis was used to determine whether differences were significant. The differences between two groups were tested with the two‐tailed Student's t test. The criteria for statistical significance was *p* < 0.05.

## Conflict of Interest

The authors declare no conflict of interest.

## Author Contributions

H.Z., S.L., Q.Q., and Z.X. contributed equally to this work. X.J., L.Z., and H.Z. conceived and designed the experiments. H.Z., S.L., Q.Q., Z.X., Y.Q., Y.W., J.W., Z.D., Y.L., R.T., S.Y., S.H., Z.C., S.H., Z.C., and W.H. performed the experiments. H.Z., S.L., Q.Q., Z.X., Y.Q., Y.W., J.W., Z.D., Y.L., R.T., S.Y., W.H., X.Y., Y.W., L.Z. and X.J. analyzed the experimental data. W.H., Y.W., L.Z., and X.J. provided regent. H.Z., X.Y., L.Z., and X.J. wrote the manuscript. All authors read and approved the manuscript.

## Supporting information

Supporting Information

## Data Availability

The data that support the findings of this study are available in the supplementary material of this article.
