# Supplementary material for: Genetic and Pharmacological Inhibition of Astrocytic Mysm1 Alleviates Depressive‐Like Disorders by Promoting ATP Production
Source: Adv Sci (Weinh). 2022 Nov 22;10(1):2204463. doi: 10.1002/advs.202204463 (PMC9811473; doi:10.1002/advs.202204463)
Supplement: Supplementary file 1 — Supporting Information [file ADVS-10-2204463-s001.pdf]

ADVANCED SCIENCE
Open Access

Supporting Information

for *Adv. Sci.*, DOI 10.1002/advs.202204463

Genetic and Pharmacological Inhibition of Astrocytic Mysm1 Alleviates Depressive-Like Disorders by Promoting ATP Production

Heyang Zhang, Shuirong Liu, Qiaozhen Qin, Zhenhua Xu, Yannv Qu, Yadi Wang, Jianing Wang, Zhangzhen Du, Shanshan Yuan, Shunming Hong, Zhilin Chang, Wenyan He, Xinlong Yan, Yiran Lang, Rongyu Tang, Yan Wang, Lingling Zhu* and Xiaoxia Jiang**

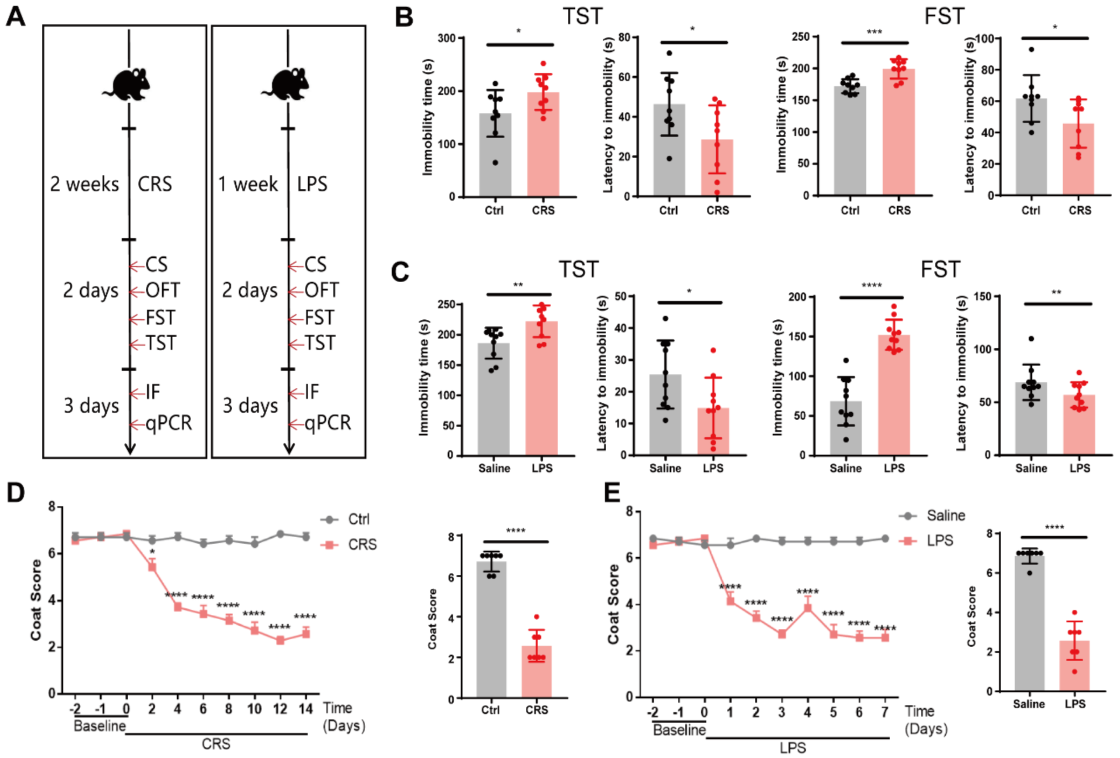

Figure S1. CRS- and LPS-treated mice show depression-like and anxiety-like behaviors

(A) Schematic illustrating depression modeling in mice. (B) Results from the Ctrl and CRS groups that were tested by TST and FST. (C) Results from the saline and LPS groups that were tested by the TST and FST. (D) CRS mice showed lower coat scores than control mice. (E) LPS-treated mice showed lower coat scores than control mice. Data represent the mean ± SEM; ns, not significant; *$p < 0.05$, **$p < 0.01$, ***$p < 0.001$, and ****$p < 0.0001$.

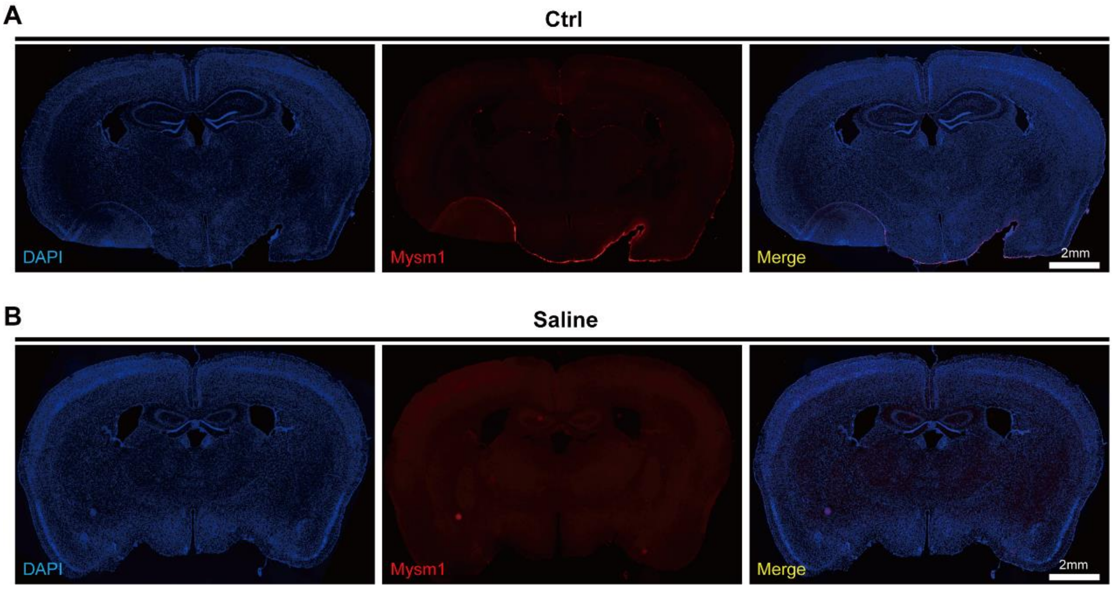

Figure S2. The expression of Mysm1 in brain slices from normal and saline-treated C57BL/6N mice

(A)Representative immunofluorescent staining of Mysm1 expression in coronal brain sections from normal C57BL/6N mice. Scale bar, 2 mm. (B)Representative immunofluorescent staining of Mysm1 expression in coronal brain sections from saline-treated C57BL/6N mice. Scale bar, 2 mm.

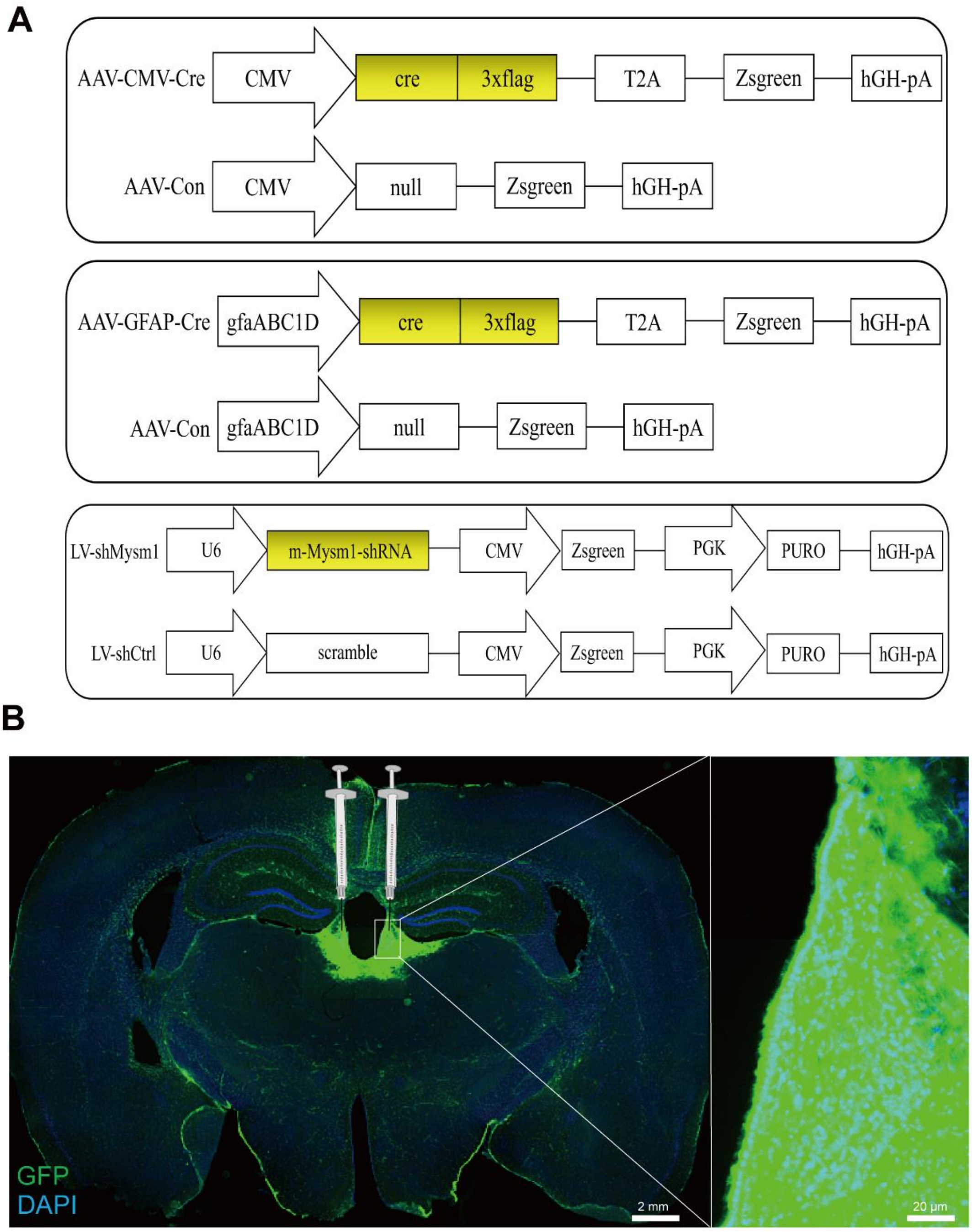

Figure S3. Schematic of the vectors and the injection in the MHb

(A) Diagrams of the AAV and LV vectors used in the studies. (B) Representative image of GFP expression in the MHb 3 weeks after injection. Scale bars, 2 mm, 20 μm.

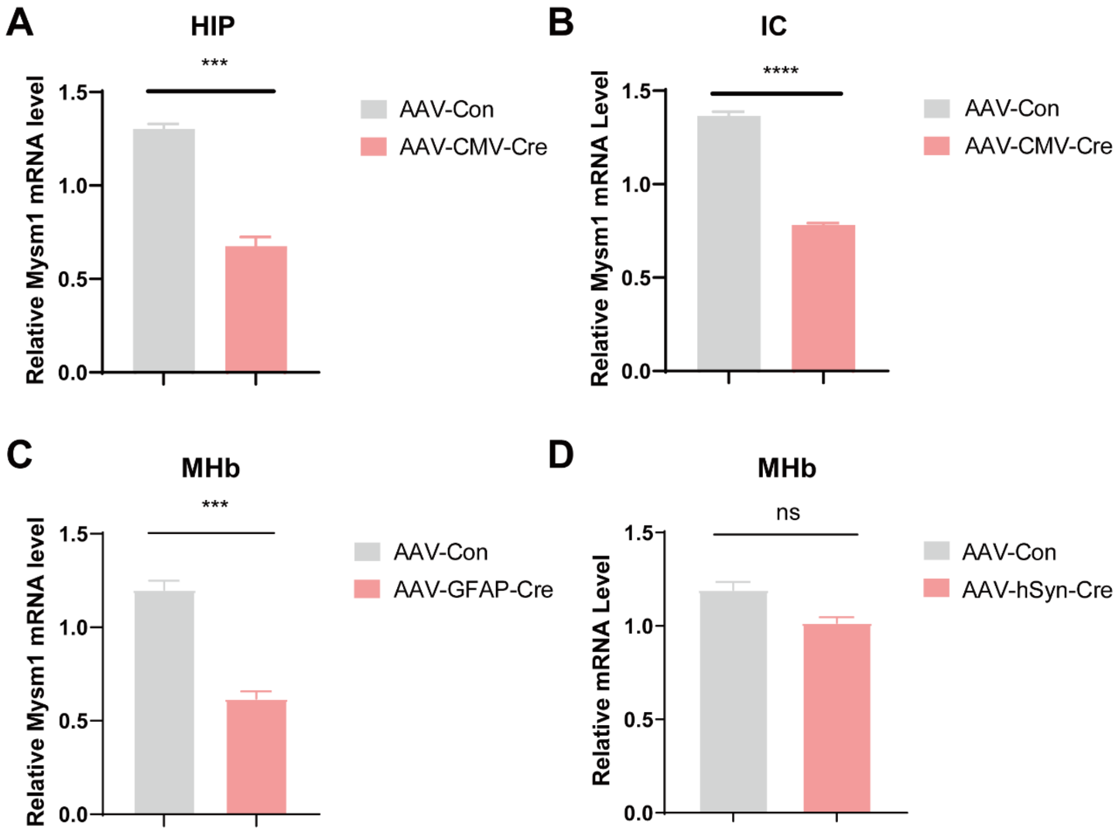

Figure S4. Mysm1 knockdown efficiency in HIP, IC and MHb.

(A) qRT-PCR results of Mysm1 in the HIP of AAV-CMV-Cre knockdown depressed mice. (B) qRT-PCR results of Mysm1 in the IC of AAV-CMV-Cre knockdown depressed mice. (C) qRT-PCR results of Mysm1 in the MHb of AAV-GFAP-Cre knockdown depressed mice. (D) qRT-PCR results of Mysm1 in the MHb of AAV-hSyn-Cre knockdown depressed mice. Data represent the mean ± SEM; ns, not significant; ***$P < 0.001$ and ****$P < 0.0001$.

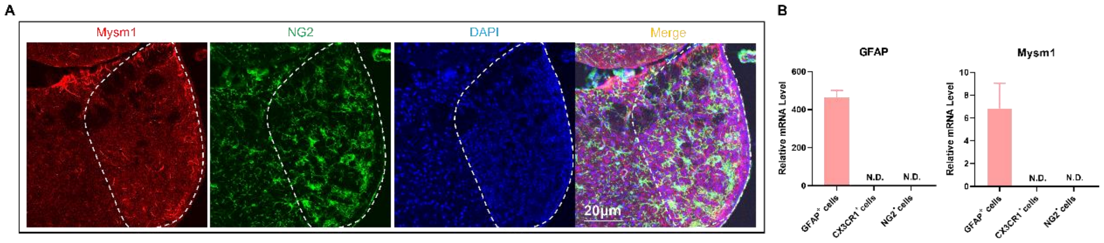

Figure S5. Mysm1 has no obvious expression in pro-oligodendrocytes and is detected in sorted GFAP positive cells.

(A) Immunofluorescent staining of Mysm1 and NG2 in coronal brain sections and higher-magnification images of the MHb. Scale bar, 20 µm. (B) qRT-PCR results of Mysm1 expression in astrocytes (GFAP), microglia (CX3CR1) and pro-oligodendrocytes (NG2) after flow sorting.

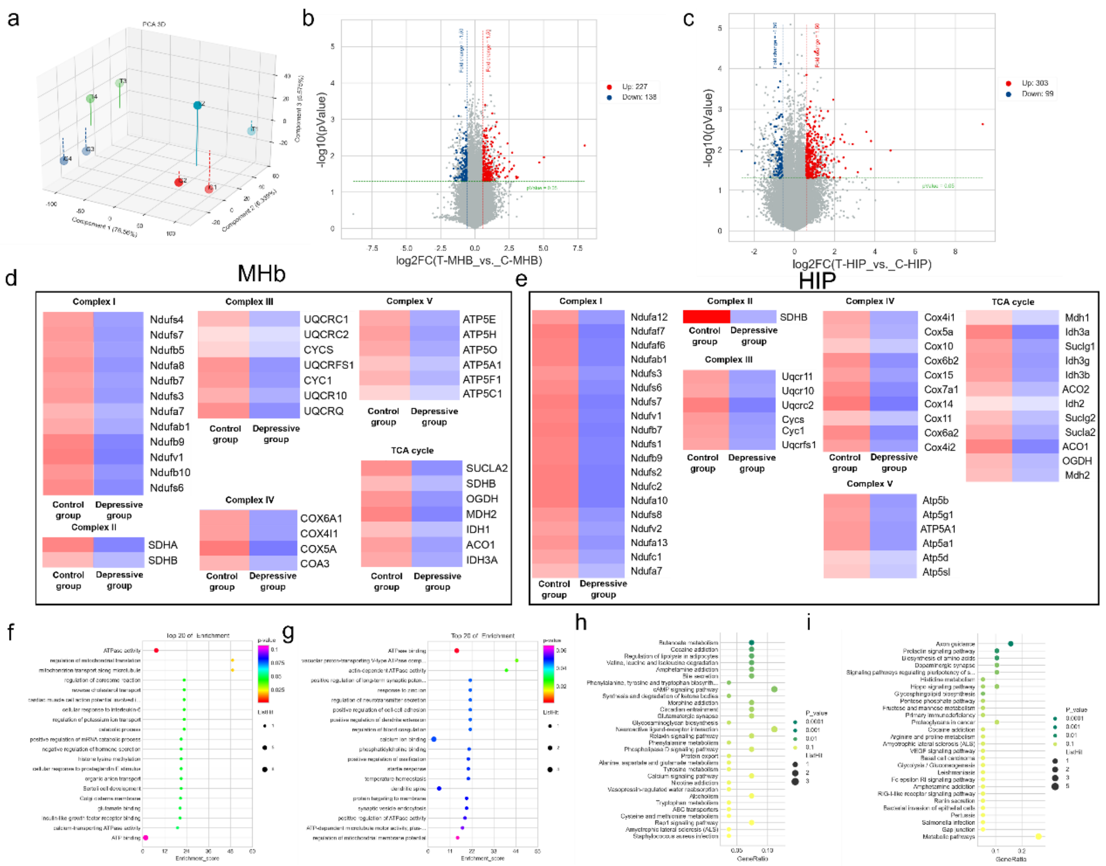

Figure S6. RNA sequencing result of MHb and HIP in depressive mice

(A) PCA plot using the top quartile of the most variable genes across the samples. (B-C) Volcano plot of differentially expressed genes between the control and depressive groups. The X-axis represents the fold change, while the Y-axis indicates the significance of differential expression. The gray points indicate unigenes with no significant change (P < 0.05, false discovery rate (FDR) q < 0.05), while the red points and the blue points indicate up- and downregulated unigenes (P < 0.05, FDR q < 0.05), respectively. (D-E) Heatmap of control and depressive mice in the MHb and HIP. The heatmap was generated based on FPKM of RNA-seq data, which represents the unigene expression levels. The red region indicates high expression levels in response to a challenge with legend stimulation. The blue region indicates low expression levels in response to a challenge with legend stimulation. (F-G) Gene ontology enrichment gene number is defined as the number of target genes in MHb and HIP. The rich factor is defined as the number of target genes divided by the number of all the genes in each term. The number of GO target genes, p value and rich factor are indicated in the scatterplot. (H-I) KEGG pathway enrichment gene number is defined as the number of target genes in MHb and HIP. KEGG pathways were calculated, and those with a p value < 0.05 were defined as significant.

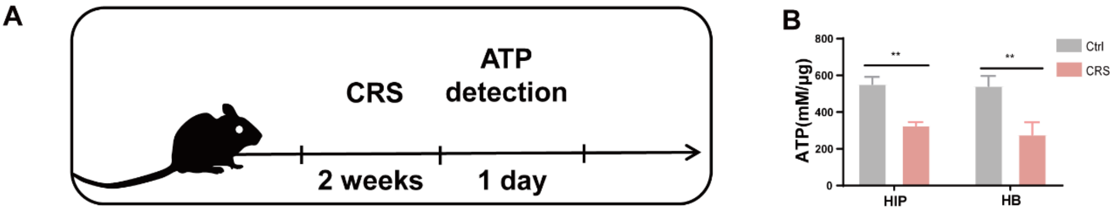

Figure S7. ATP levels in the hippocampus and habenula of depressed mice were significantly decreased

(A) Schematic illustrating ATP detection in depressed mice. (B) Quantification of HIP and HB ATP in control or CRS mice. Data represent the mean ± SEM; ns, not significant; $**p < 0.01$.

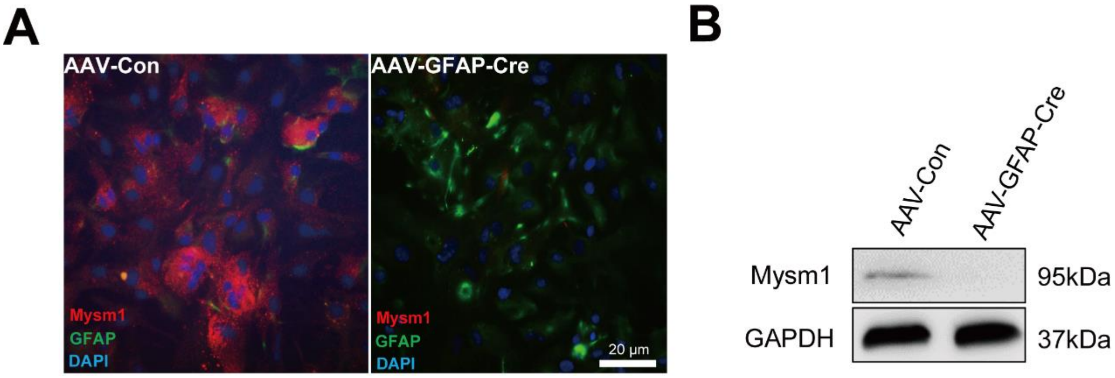

Figure S8. Decreased expression of Mysm1 in astrocytes from Mysm1$^{fl/fl}$ mouse with AAV-GFAP-Cre transduction

Immunofluorescence staining (A) and western blot (B) showing efficient knockdown of Mysm1 in astrocytes from Mysm1$^{fl/fl}$ mice after transduced with AAV-GFAP-Cre virus. Scale bars, 20 μm.

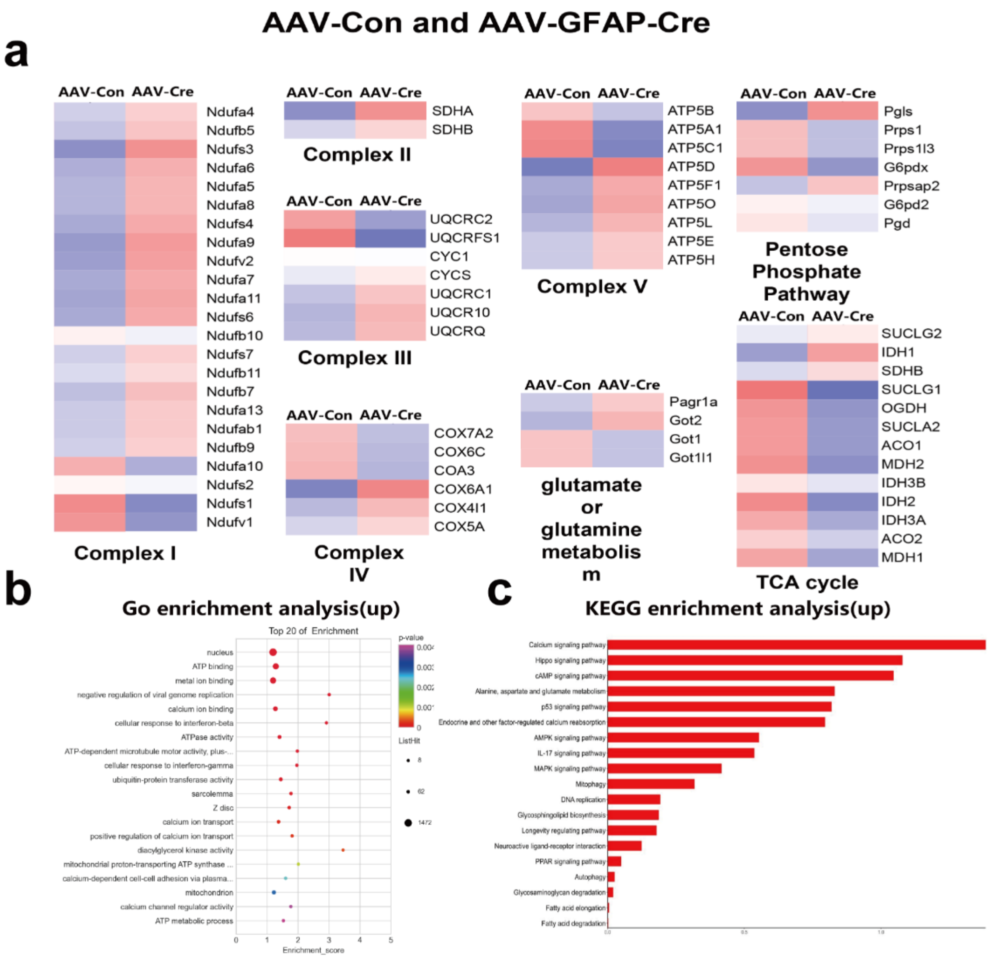

Figure S9. Astrocytic Mysm1 knockdown enhances mitochondrial metabolism and the TCA cycle

(A) Heatmap showing hierarchical clustering of genes upregulated (red) or downregulated (blue) in astrocytes. (B) Gene ontology enrichment gene number is defined as the number of target genes in each term. The rich factor is defined as the number of target genes divided by the number of all the genes in each term. The number of GO target genes, p value and rich factor are indicated in the scatterplot. (C) KEGG pathway enrichment gene number is defined as the number of target genes in each pathway. KEGG pathways were calculated, and those with a p value < 0.05 were defined as significant.
